# Supplementary figures and images for: Virulence and community dynamics of fungal species with vertical and horizontal transmission on a plant with multiple infections
Source: PLoS Pathog. 2021 Jul 15;17(7):e1009769. doi: 10.1371/journal.ppat.1009769 (PMC8315517; doi:10.1371/journal.ppat.1009769)

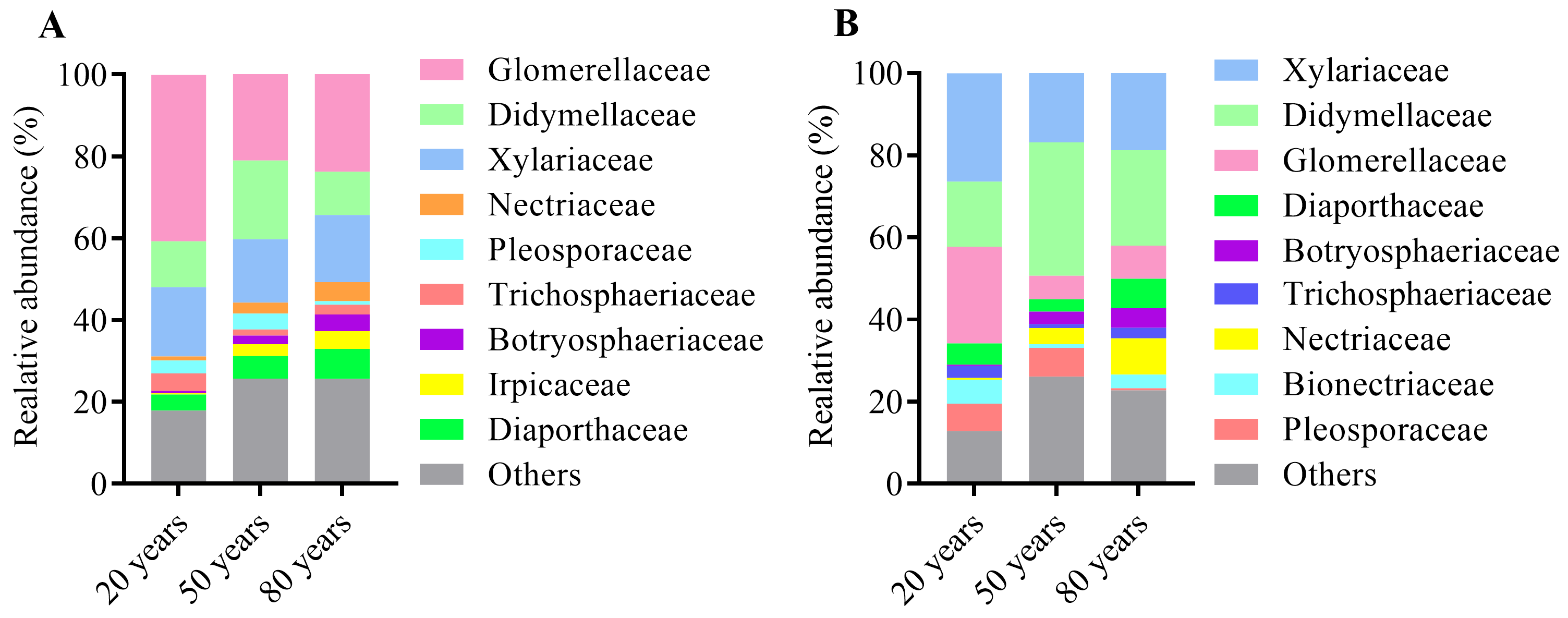

Supplement: S1 Fig — (A) Total isolated fungi. (B) The fungi verified to be pathogenic to A. adenophora. (TIF) [file ppat.1009769.s001.tif]

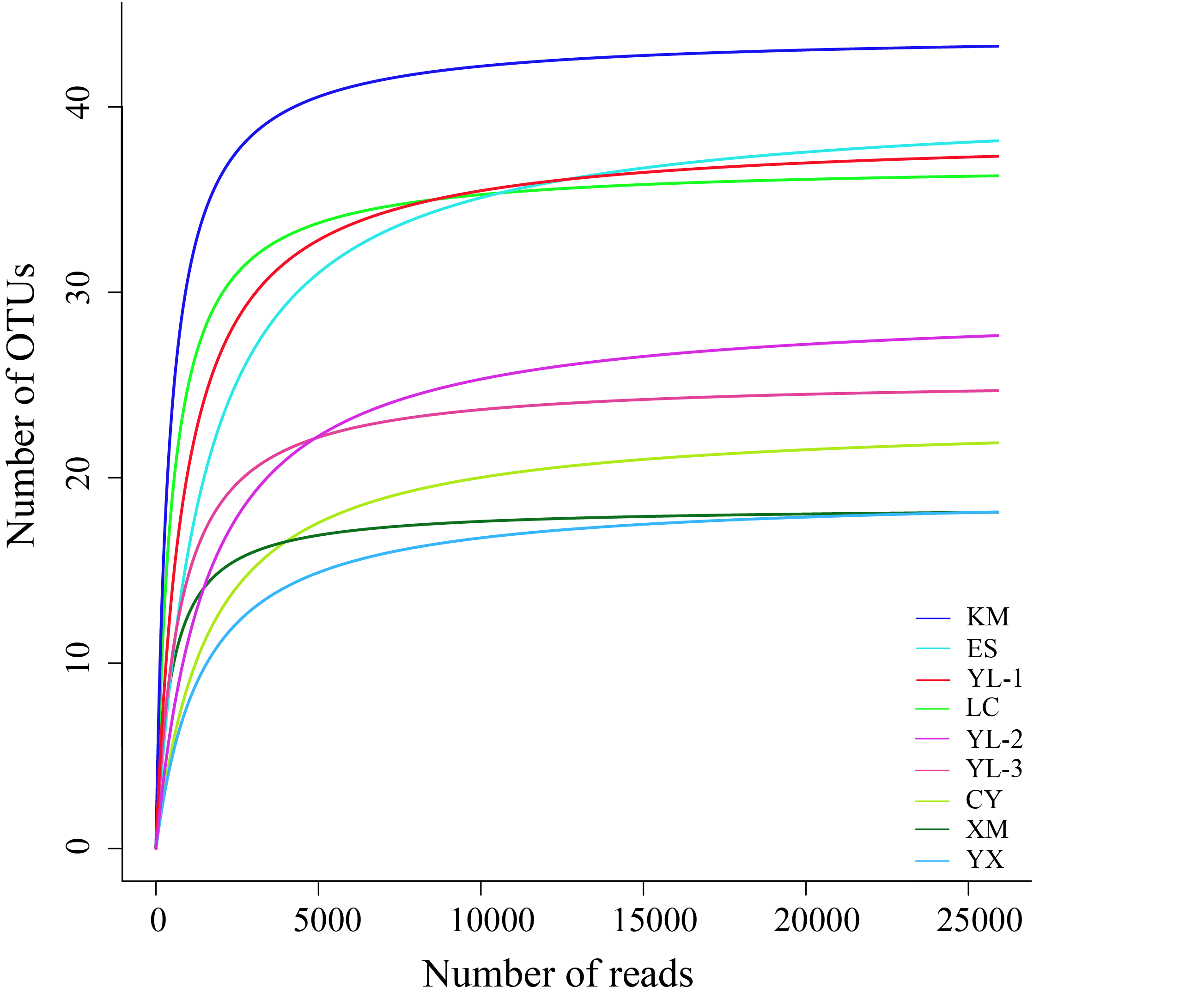

Supplement: S2 Fig — The geographic information for the A. adenophora population is detailed in S1 Data. (TIF) [file ppat.1009769.s002.tif]

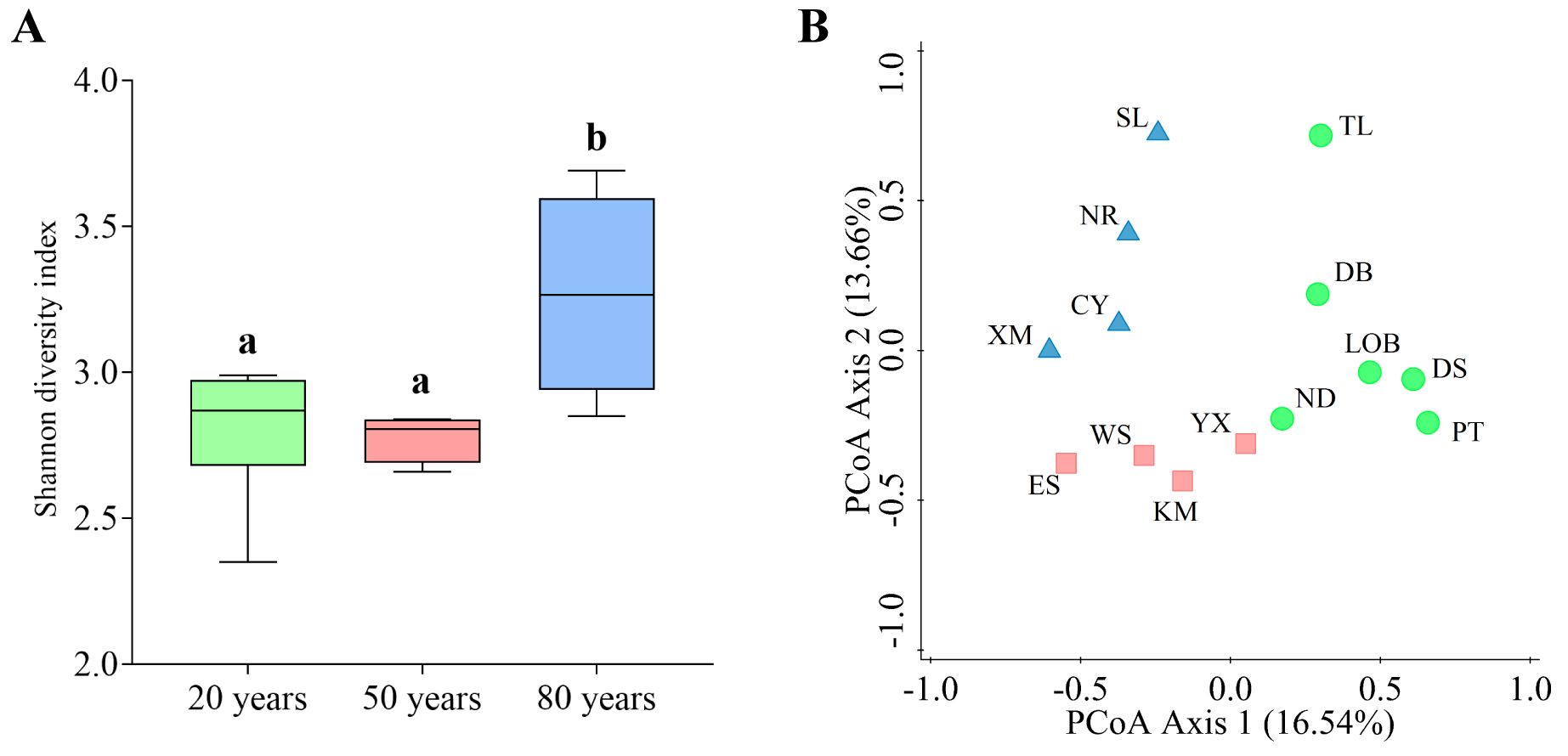

Supplement: S3 Fig — Diversity (A) and structure (B) of fungal pathogens at the geographic site level. (A) Nonparametric analysis with the Mann-Whitney U test was performed to show that the Shannon diversity index difference was significant among invasion times by different lowercase letters (p < 0.05). (B) Principal coordinate analysis (PCoA) shows the similarity of pathogenic fungal communities among A. adenophora populations. Each spot represents one geographic site (for details, see Table 2). Percentages of total explained variation by the PCoA axes in each plot are given in parentheses. (TIF) [file ppat.1009769.s003.tif]

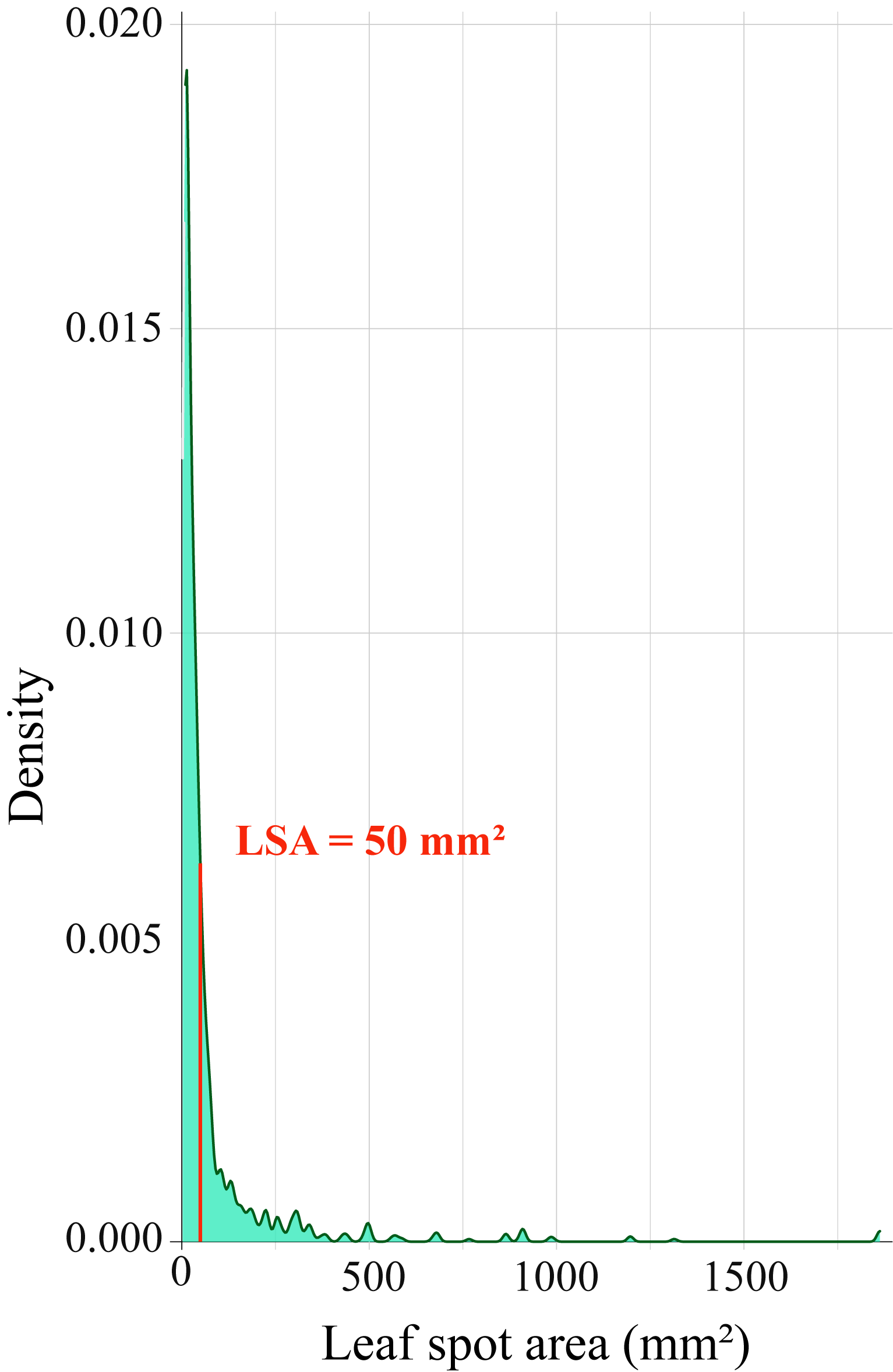

Supplement: S4 Fig — (TIF) [file ppat.1009769.s004.tif]

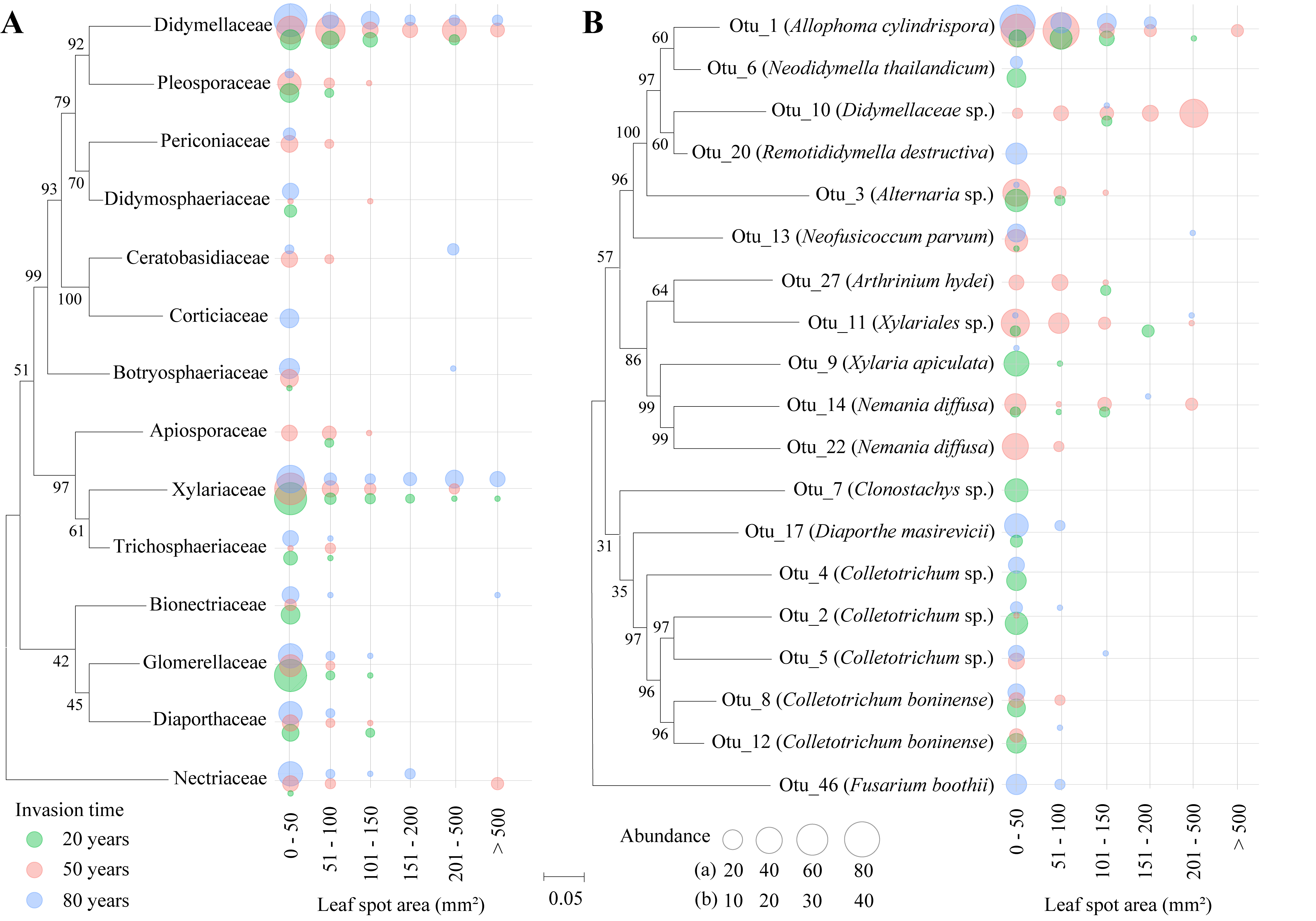

Supplement: S5 Fig — Abundance of fungal pathogens with different virulence to A. adenophora at the family level (A) and OTU level (B). (A) Families with relative abundances greater than 1% are shown, and the 14 relatively abundant families account for 86.2% of the total abundance. (B) OTUs with relative abundances greater than 1% are shown, and the 19 relatively abundant OTUs account for 50.1% of the total. The families (or OTUs) are clustered according to phylogenetic position estimated by maximum likelihood with 1000 replicates, and the bootstrap percentages are indicated at the branch node. According to the density distribution of the virulence of fungal pathogens to A. adenophora, 5 virulence ranges (LSA ≤ 50 mm2, 50 < LSA ≤ 100 mm2, 100 < LSA ≤ 150 mm2, 150 < LSA ≤ 200 mm2, 200 < LSA ≤ 500 mm2 and LSA > 500 mm2) were selected to display the data. The scale bar represents the genetic distance. (TIF) [file ppat.1009769.s005.tif]

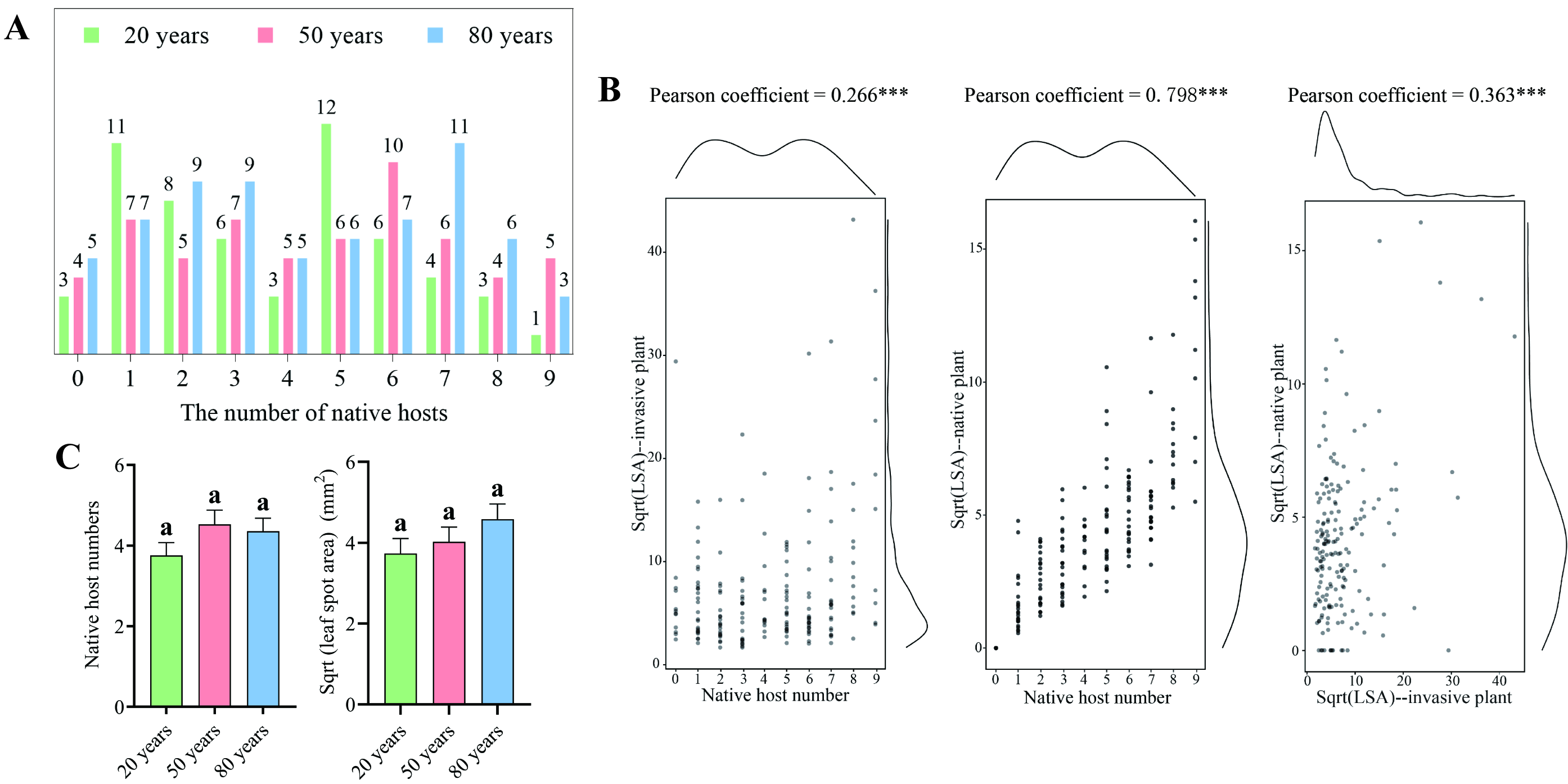

Supplement: S6 Fig — (A) Host range distribution of 184 pathogenic fungi from different invasion times. The number above each bar represents the number of isolates. (B) Pearson correlations among native host number, virulence to A. adenophora and average virulence to native plants of fungal pathogens (n = 184). (C) Average host range and virulence to native plants of 184 selected fungi at different invasion times (20 years: n = 57; 50 years: n = 59; 80 years: n = 68). Nonparametric analysis with the Mann-Whitney U test was performed to test the significance of the differences, and different lowercase letters indicate significant differences (p < 0.05). The error bar represents the standard error. (TIF) [file ppat.1009769.s006.tif]

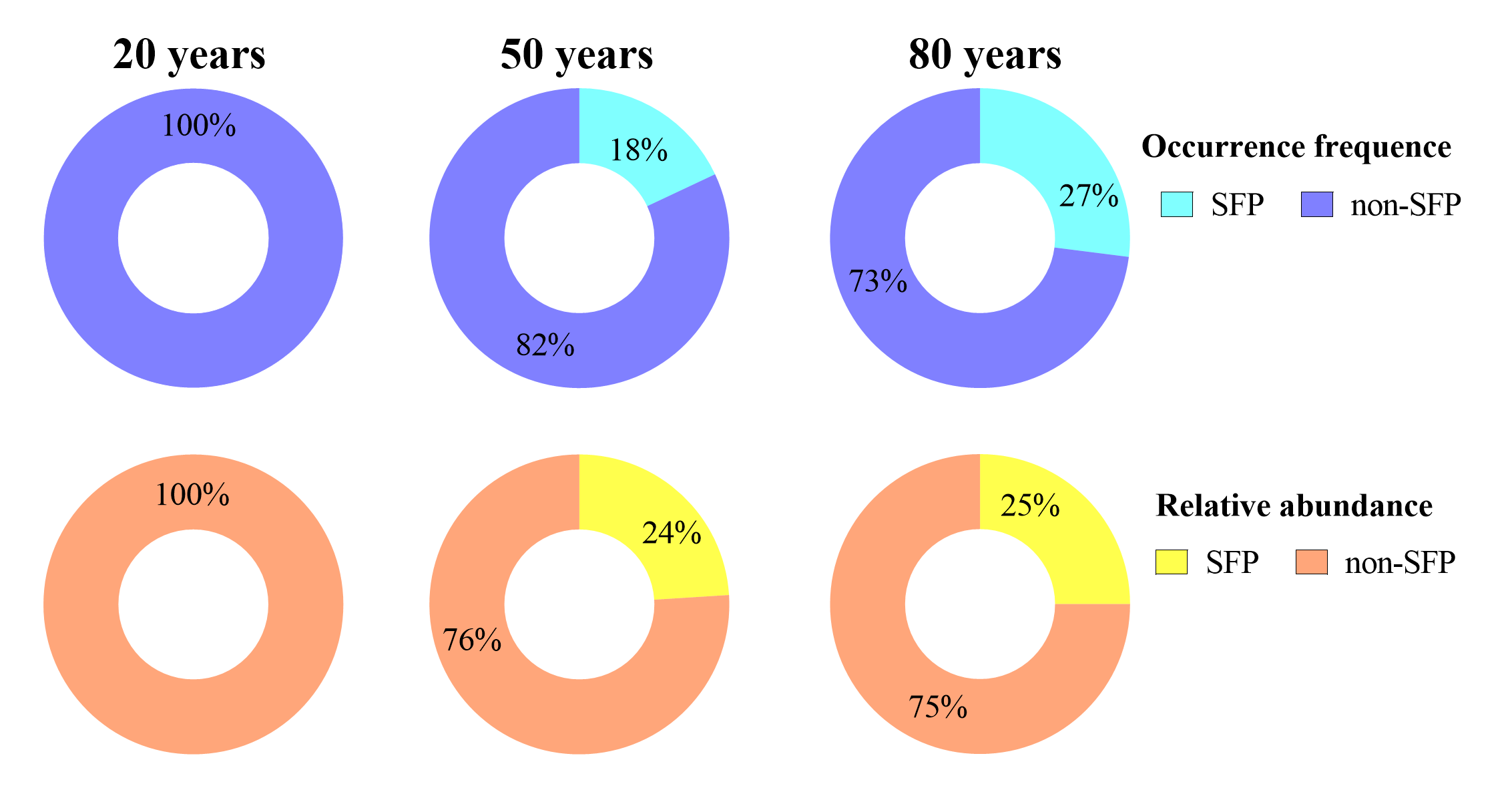

Supplement: S7 Fig — (TIF) [file ppat.1009769.s007.tif]

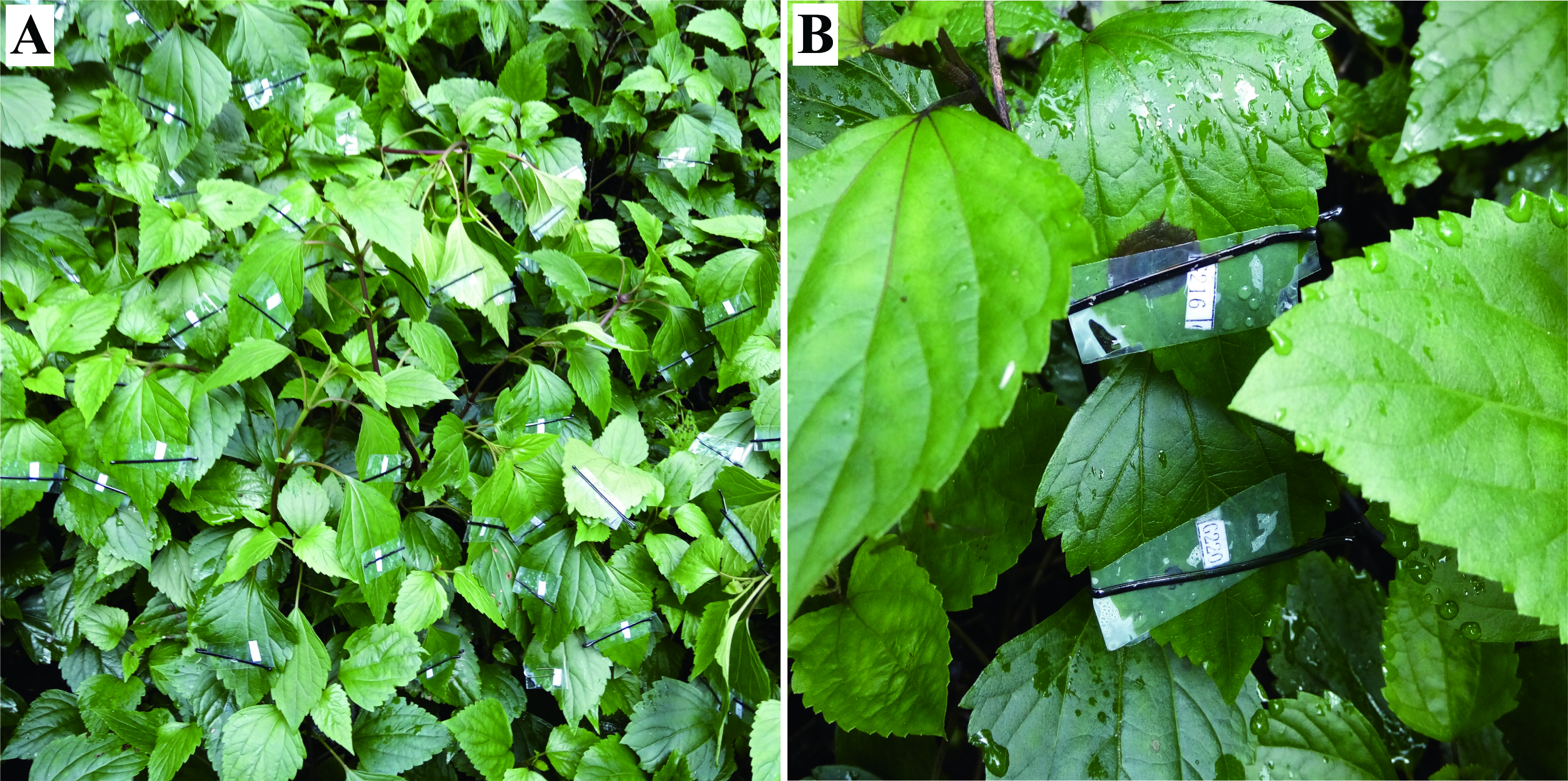

Supplement: S8 Fig — (A) Examples of inoculated individuals of A. adenophora in the field. (B) Examples of symptoms developed by A. adenophora in response to different fungal strains. (TIF) [file ppat.1009769.s008.tif]
