# Supplementary material for: Virulence and community dynamics of fungal species with vertical and horizontal transmission on a plant with multiple infections
Source: PLoS Pathog. 2021 Jul 15;17(7):e1009769. doi: 10.1371/journal.ppat.1009769 (PMC8315517; doi:10.1371/journal.ppat.1009769)
Supplement: Attachment — Submitted filename: PPATHOGENS-D-21-00073_reviewer.docx [file ppat.1009769.s010.docx]

PLOS Pathogens

Virulence evolution of multiple infections with vertically and horizontally transmitted fungal species in a natural plant system

--Manuscript Draft--

| Manuscript Number: | PPATHOGENS-D-21-00073 |
| --- | --- |
| Full Title: | Virulence evolution of multiple infections with vertically and horizontally transmitted fungal species in a natural plant system |
| Short Title: | Multiple infections on invasive plant |
| Article Type: | Research Article |
| Section/Category: | Plant Pathogens |
| Keywords: | invasive plant; multiple infections of different species; virulence evolution; transmission mode; seedborne fungi; primary pathogen |
| Abstract: | The virulence evolution of multiple infections of parasites from the same species has been modelled widely in evolution theory. However, experimental studies on this topic remain scarce, particularly regarding multiple infections by different parasite species. Here we employed the invasive plant Ageratina adenophora to verify the predictions made by the model. We observed that A. adenophora was a highly susceptible host to diverse foliar pathogens with mixed vertical and horizontal transmission within leaf spots. The pathogen community structure at the leaf spot level was determined by transmission mode. Over time, the pathogen community decreased in diversity; meanwhile, the vertically transmitted pathogens exhibited decreased virulence to the host A. adenophora, but the horizontally transmitted pathogens exhibited increased virulence to the host. Our results demonstrate that the predictions of classical models are still valid in a complex environment. Moreover, we propose that it is very important to determine whether the primary foliar pathogen of a given plant host is relevant to seedborne fungi, as this characteristic is an important factor in understanding pathogen-host interactions. |
| Additional Information: |  |
| Question | Response |
| Financial Disclosure  Enter a financial disclosure statement that describes the sources of funding for the work included in this submission. Review the [submission guidelines](http://journals.plos.org/plospathogens/s/submission-guidelines#loc-financial-disclosure-statement) for detailed requirements. View published research articles from [PLOS Pathogens](http://journals.plos.org/plospathogens/) for specific examples.  This statement is required for submission and will appear in the published article if the submission is accepted. Please make sure it is accurate. | HZ acquired finacially supports from the National Natural Science Foundation of China (grant nos. 31770585 and 31360153). The funders had no role in study design, data collection and analysis, decision to publish, or preparation of the manuscript. |

| Unfunded studies  Enter: The author(s) received no specific funding for this work.  Funded studies  Enter a statement with the following details:   - Initials of the authors who received each award - Grant numbers awarded to each author - The full name of each funder - URL of each funder website - Did the sponsors or funders play any role in the study design, data collection and analysis, decision to publish, or preparation of the manuscript? - NO - Include this sentence at the end of your statement: The funders had no role in study design, data collection and analysis, decision to publish, or preparation of the manuscript. - YES - Specify the role(s) played. |  |
| --- | --- |
| * typeset |  |
| Competing Interests  Use the instructions below to enter a competing interest statement for this submission. On behalf of all authors, disclose any [competing interests](http://journals.plos.org/plospathogens/s/competing-interests) that could be perceived to bias this work—acknowledging all financial support and any other relevant financial or non- financial competing interests.  This statement will appear in the published article if the submission is accepted. Please make sure it is accurate. View published research articles from [PLOS Pathogens](http://journals.plos.org/plospathogens/) for specific examples. | The authors have declared that no competing interests exist. |

| NO authors have competing interests  Enter: The authors have declared that no competing interests exist.  Authors with competing interests  Enter competing interest details beginning with this statement:  I have read the journal's policy and the authors of this manuscript have the following competing interests: [insert competing interests here] |  |
| --- | --- |
| * typeset |  |
| Data Availability  Authors are required to make all data underlying the findings described fully available, without restriction, and from the time of publication. PLOS allows rare exceptions to address legal and ethical concerns. See the [PLOS Data Policy](http://journals.plos.org/plospathogens/s/data-availability) and [FAQ](http://journals.plos.org/plospathogens/s/data-availability#loc-faqs-for-data-policy) for detailed information.  A Data Availability Statement describing where the data can be found is required at submission. Your answers to this question constitute the Data Availability Statement and will be published in the article, if accepted. | Yes - all data are fully available without restriction |
| Important: Stating ‘data available on request from the author’ is not sufficient. If your data are only available upon request, select ‘No’ for the first question and explain your exceptional situation in the text box. |  |
| Do the authors confirm that all data underlying the findings described in their manuscript are fully available without restriction? |  |
| Describe where the data may be found in full sentences. If you are copying our sample text, replace any instances of XXX with the appropriate details.   - If the data are held or will be held in a | All relevant data are within the manuscript and its Supporting Information files. |

| public repository, include URLs, accession numbers or DOIs. If this information will only be available after acceptance, indicate this by ticking the box below. For example: All XXX files are available from the XXX database (accession number(s) XXX, XXX.).   - If the data are all contained within the manuscript and/or Supporting Information files, enter the following: All relevant data are within the manuscript and its Supporting Information files. - If neither of these applies but you are able to provide details of access elsewhere, with or without limitations, please do so. For example:   Data cannot be shared publicly because of [XXX]. Data are available from the XXX Institutional Data Access / Ethics Committee (contact via XXX) for researchers who meet the criteria for access to confidential data.  The data underlying the results presented in the study are available from (include the name of the third party and contact information or URL).   - This text is appropriate if the data are owned by a third party and authors do not have permission to share the data.   * typeset |  |
| --- | --- |
| Additional data availability information: |  |

manuscript	[Click here to access/download;Manuscript;main text(1-11).docx](https://www.editorialmanager.com/ppathogens/download.aspx?id=1547694&guid=43c869f6-cea2-4c43-ac7d-05f26c946df2&scheme=1)

## 1 Title Page

2

## 3 Virulence evolution of multiple infections with vertically and horizontally

4 **transmitted fungal species in a natural plant system**

5

6	**Running title:** Multiple infections on invasive plant

7

8 ***Author Affiliations*:**
9 Kai Fanga,b†, Jie Zhoua†, Lin Chena,b, Yu-Xuan Lia, Ai-Ling Yanga, Xing-Fan Donga, Han-Bo
10 Zhanga,b*
11 aState Key Laboratory for Conservation and Utilization of Bio-Resources in Yunnan, Yunnan
12 University, Kunming 650091, China; bSchool of Ecology and Environmental Science, Yunnan
13 University, Kunming 650091, China.

14

15 ***Corresponding Author*:** Han-Bo Zhang, State Key Laboratory for Conservation and Utilization of
16 Bio-Resources in Yunnan, Yunnan University, Kunming 650091, China. Tel: +86-0871-65031412;
17 Email address: [zhhb@ynu.edu.cn.](mailto:zhhb@ynu.edu.cn)

18

19	†Equal contributors

20

## 21 Abstract

22 The virulence evolution of multiple infections of parasites from the same species has been
23 modelled widely in evolution theory. However, experimental studies on this topic remain scarce,
24 particularly regarding multiple infections by different parasite species. Here we employed the
25 invasive plant *Ageratina adenophora* to verify the predictions made by the model. We observed
26 that *A. adenophora* was a highly susceptible host to diverse foliar pathogens with mixed vertical
27 and horizontal transmission within leaf spots. The pathogen community structure at the leaf spot
28 level was determined by transmission mode. Over time, the pathogen community decreased in
29 diversity; meanwhile, the vertically transmitted pathogens exhibited decreased virulence to the
30 host *A. adenophora*, but the horizontally transmitted pathogens exhibited increased virulence to
31 the host. Our results demonstrate that the predictions of classical models are still valid in a
32 complex environment. Moreover, we propose that it is very important to determine whether the
33 primary foliar pathogen of a given plant host is relevant to seedborne fungi, as this characteristic is
34 an important factor in understanding pathogen-host interactions.

35

## 36 Author Summary

37 To date there is a lack of experimental evolution studies on virulence evolution in the
38 context of multiple infections by different species with both vertical and horizontal transmission
39 modes. Here we employed a naturally occurred host–parasite system, the invasive plant *Ageratina*
40 *adenophora* and its foliar pathogens, to verify theoretical predictions. We observed that *A.*
41 *adenophora* was a highly susceptible host to diverse foliar pathogens with mixed vertical and
42 horizontal transmission within leaf spots. The transmission mode determined the pathogen
43 community structure within leaf spots. Over time, the pathogen community decreased in diversity;
44 meanwhile, the vertically transmitted pathogens exhibited decreased virulence to the host *A.*
45 *adenophora*, but the horizontally transmitted pathogens exhibited increased virulence to the host.
46 Our results demonstrate that the predictions of classical models are still valid in a complex
47 environment. Moreover, we propose that seedborne fungi are important for determining the foliar
48 pathogen-host interactions..

52

## 53 1. Introduction

54 The traditional concept of a single pathogen infecting a single host has been widely accepted
55 in plant pathology; however, a growing number of examples indicate that many plant diseases
56 are caused by multiple taxa of microbes (1, 2). Therefore, it has become increasingly
57 important to understand how multiple infections affect key components of host-pathogen
58 interactions, including within-plant virulence and pathogen accumulation, evolutionary trajectories
59 of pathogen populations, and disease dynamics (2). Classical theory predicts that
60 virulence evolution in multiple infections depends on pathogen transmission mode and population
61 dynamics of pathogen, as well as host (3). Most models assuming that parasites are transmitted
62 horizontally predict that the within-host competition of multiple infections favours increased
63 virulence of parasites; as a consequence, the increased virulence benefits the transmission of
64 parasites to other hosts, while the within-host competition excludes the weaker parasites and, in
65 turn, causes pathogen diversity to decline (3, 4). If the parasites are transmitted vertically, however,
66 natural selection benefits parasites that are less harmful to host fitness; in the case that the
67 parasites are transmitted both vertically and horizontally, the selection for low virulence becomes
68 stronger when host density increases (5).
69 Lagging behind the progress of theoretical models, however, experimental studies have
70 focused on infections consisting of multiple strains of the same species (6). For plant diseases,
71 there is still a lack of experimental evolution studies on virulence evolution in the context of
72 multiple infections by different species with mixed transmission modes, which represents the true
73 situation of pathogens associated with any given plant host in natural systems (2, 3). Alizon, de
74 Roode (3) proposed a three-step experimental evolution study on virulence. However, in the
75 laboratory or greenhouse, it is difficult to perform serial passages by inoculating multiple
76 infections of different species with distinct virulence by vertical and horizontal transmission on a
77 host. Finding a suitable naturally occurring host–parasite system is an alternative way to verify
78 theoretical predictions to date.
79 Exotic plant introduction has been an unprecedented biogeographical experiment performed
80 in nature, and compelling evidence indicates that evolution can occur in contemporary time spans
81 (7); therefore, studying invasive systems may help to elucidate how a number of ecological and
82 evolutionary processes operate at the same time (8). Exotic species
83 have frequently invaded into a novel range while leaving pathogens behind (9), however, some
84 invasive plants in non-native habitats inevitably encounter diverse local pathogens (10, 11). The
85 mechanism by which invasive plants accumulate pathogens has also been partially explained in
86 terms of ecology and evolution (10). For example, pathogen accumulation is positively correlated
87 with invasive time (12) and the expansion of the geographical scope of hosts (13); high host
88 density increases the possibility of infection (14). Both novel associations of invasive plants with
89 native pathogens and cointroduction of pathogens with invasive hosts are common (15, 16).
90 Intuitively, virulence evolution in multiple infections with mixed transmission modes naturally
91 occurs in the invasive plants over time but has not been fully characterized.
92 *Ageratina adenophora* (Compositae), a perennial herb and native to Central America, has
93 invaded more than 40 countries worldwide in tropical to temperate regions (17). Since the first
94 record in China in the 1940s, the plant has been widely distributed in the provinces of Yunnan,
95 Sichuan, Guizhou, Guangxi and Tibet, and it has continuously spread east- and northward with
96 clear invasive history records (18). There is evidence that *A. adenophora* can be infected by fungal
97 pathogens in the introduced ranges (17, 19). Our recent study indicates that *A. adenophora*
98 accumulates diverse foliar pathogens from neighbors (horizontally transmitted); however, a
99 numerically dominant pathogen (defined as OTU515 based on ITS barcoding), does not
100 occur on surrounding native plants (20). This fungus belongs to the plant pathogen-rich
101 family Didymellaceae (Ascomycota, Dothideomycetes, Pleosporales). Thus, we hypothesize that OTU515 is
102 co-spread with the host by seeds (vertically transmitted) because *A. adenophora* disperses
103 primarily by wind and water through minute asexual seeds (21). In this research, we attempted to
104 use *A. adenophora* as a model to verify the predictions made by models of multiple infections
105 with mixed transmission modes. Here we focused our multiple infections at the leaf spot level,
106 rather than the host individual level, because (i) a multiple infection within-leaf spot should
107 include both direct and indirect interactions of pathogens (2) and (ii) multiple fungal species or
108 genotypes from one species have been observed to co-occur in the same spots of *Eucalyptus* (22,
109 23); thus, multiple infections in one leaf spot should be common but remain to be characterized.

## 110 2. Results

### 111 2.1 Diversity of leaf spot fungal pathogens

112 A total of 2705 fungi (451 OTUs) were isolated from 236 leaf spots, and the disease
113 experiment screened 1149 pathogenic fungi (266 OTUs) on *A. adenophora*, phylogenetically
114 dominated by the Sordariomycete families Glomerellaceae, Xylariaceae as well as Didymellaceae (S1 Fig, S1 File). The
115 proportion of pathogenic fungi varied across families; e.g., most strains from the family
116 Didymellaceae caused lesions, but most strains from the family Glomerellaceae did not.
117 The frequencies of Pleosporaceae (Pleosporales; Dothideomycetes) and Nectriaceae (Hypocreales, Sordariomycetes) were low small but contained
118 high proportions of pathogens. At the OTU level, the common pathogens were from the genera
119 *Allophoma*, *Alternaria*, *Colletotrichum*, *Xylaria*, and *Neofusicoccum*; in particular, the	most common
120 pathogen OTU1 (*Allophoma cylindrispora*, Didymellaceae) was at high levels in three invasive
121 areas (Fig 1a).
122 In total, 212 (89.8%) leaf spots successfully harvested pathogens, with an average of 5.4
123 pathogen OTUs per spot being observed. Among these leaf spots, 164 contained > 2 pathogens of
124 different fungal species and one leaf spot contained >2 genotypes from a single species (Fig 1b,
125 S1 File). Subsequently, these leaf spots were considered multiple infections and were employed to
126 analyze the virulence evolution and community dynamics of pathogens.

### 127 2.2 Seedborne foliar pathogens of *A. adenophora*

128 The bulked seeds of nine *A. adenophora* populations were subjected to high-throughput
129 sequencing, and 179 OTUs (591561 reads) were identified, phylogenetically belonging to 3 phyla,
130 27 families and 43 genera (S1 File; for rarefaction curves, see S2 Fig). 17 overlapping OTUs overlapped
131 between foliar and seedborne samples, accounting for 78.3%
132 (462980 reads) and 11.5% (311 isolates) of the two fungal libraries, respectively (Fig 2a). Among
133 these isolates of foliar fungi, 291 were pathogenic to *A. adenophora* (including 13 OTUs,
134 accounting for 25.3% of the total fungal pathogens); the shared foliar pathogen OTU1 accounted
135 for 21.5% of seedborne fungal reads and 15.6% of leaf spot pathogens (Fig 2, S1 File).

### 136 2.3 Diversity and structure of fungal pathogens across invasive history at the macroscale and

137 **microscale level**
138 At the macroscale level (geographic site), the pathogen diversity at 80 years was the highest
139 (S3 Fig, panel a), and pathogen communities were clustered with invasive time (F = 2.0, *p* = 0.002,
140 14.3% explanatory variables (EV), 7.1% adjusted explained variation (AEV) (S3 Fig, panel b).
141 However, at the microscale level (leaf spot) with multiple infections, pathogen diversity decreased
142 over time (Fig 3a). Interestingly, pathogen communities were clustered by fungal transmission
143 mode (seedborne pathogens (SPs) vs. non-seedborne pathogens (non-SPs)) (F = 16.7, *p* = 0.002,
144 7.4% EV, 6.9% AEV); 79 leaf spots contained SP pathogens, and most of them were separated
145 from those leaf spots only containing non-SPs (Fig 3b). Seedborne pathogens (SPs) in leaf spots
146 were marginally more diverse than non-seedborne pathogens (non-SPs), but both decreased with
147 invasive time (Fig 3c). When we pooled all 13 SP OTUs for comparison, we found that they were
148 slightly elevated in old sites (especially at 50 years); in contrast, the non-SP OTUs were elevated
149 in the leaf spots of newly established populations (Fig 3d).
150 **2.4 Fungal virulence evolution on host *A. adenophora***
151 Nearly 71% of the total pathogens were weakly virulent to host *A. adenophora* (leaf spot area
152 (LSA) < 50 mm^2^) (S4 Fig). Most Glomerellaceae were weakly virulent (LSA ≤ 50 mm^2^), while
153 Didymellaceae and Xylariaceae contained the most highly virulent strains (LSA > 200 mm^2^) (S5
154 Fig). The within-spot virulence variation increased linearly with pathogen diversity (Fig 4a). On
155 average, strains were less virulent but had higher virulence variation within spots in 20-year areas
156 than in old areas (especially 50-year areas) (Fig 4b). When separately analysing SPs and non-SPs,
157 SPs showed significantly higher virulence to	*A. adenophora*	than	non-SPs; SPs virulence
158 decreased, but non-SPs increased, to *A. adenophora* over time (Fig 4c).

### 159 2.5 Fungal virulence evolution on native plants

160 Among 184 fungal pathogens (including 28 SPs and 156 non-SPs) of *A. adenophora*, only 12
161 isolates were avirulent to any of the tested nine native species (S6 Fig, panel a). There were
162 significantly positive correlations among fungal pathogens’ virulence to *A. adenophora* and
163 virulence to native species and pathogenic range (S6 Fig, panel b). Overall, these pathogens had
164 no change in the host range or virulence to native plants over time (S6 Fig, panel c). In addition,
165 both SPs and non-SPs were more virulent to *A. adenophora* than to native plants (Fig 5a).
166 However, SPs were less virulent to native plants and had a narrower host range than non-SPs, and
167 this pattern also did not change over time (Fig 5b, c).

## 168 3. Discussion

169 Understanding how the pathogen community associated with invasive plants changes over
170 time has been an increasing concern of invasive biologists (12, 15, 16). We observed that the
171 invasive *A. adenophora* was a highly susceptible host to phylogenetically diverse foliar pathogens
172 in the introduced range and, in particular, 165 of investigated leaf spots (77.8%) showed multiple
173 infections (Fig 1b). Further experiments indicated that 25.3% of foliar pathogens were seedborne
174 (Fig 2a, S1 File). Therefore, this invasive plant system provided an ideal opportunity to study
175 virulence evolution of multiple infections with mixed vertical and horizontal transmissions, a
176 significant evolutionary theory that is yet to be verified by experimental studies in plant disease

177	(3).

178 In general, the recently introduced hosts support uniformly low pathogen richness, whereas
179 longer-established hosts support a greater average number of pathogen species (12). Accordingly,
180 *A. adenophora* pathogen diversity (S3 Fig, panel a) increased over time at the macroscale level
181 (geographic site). However, our important finding was that at the microscale level (the leaf spot)
182 where multiple infections are considered, this trend was reversed, with new invaders harbouring
183 more diverse pathogens than older invaders (Fig 3a). More interestingly, pathogen communities at
184 the leaf spot level were clustered by pathogen transmission modes (SP vs non-SP) (Fig 3b). The
185 pathogen assembly of multiple infections is affected by biotic and abiotic factors (2, 24). Our data
186 revealed that seedborne fungi (vertical transmission) can affect the subsequent colonization of
187 other horizontally obtained pathogens on leaf spots. The reason for this phenomenon may be
188 relevant to the priority effects that SPs systematically grow within hosts to produce, as the primary
189 symbiont hypothesis (PSH) indicated (25). Our previous report indicated that members of the
190 family Didymellaceae (the primary SP fungi in this case, see Fig 2) can asymptomatically grow
191 within healthy leaves (26) and stems (27), and their growth is stimulated by leaf extracts (26).
192 That manner of growth explains why nearly 48% of leaf spots with multiple infections contain SP
193 fungi (Fig 3d, S1 File).
194 For horizontally transmitted pathogens, classical theoretical models regarding multiple-strain
195 infections from one species predict that competition exclusion within the host favours pathogenic
196 strains with higher virulence which, in turn, causes lower pathogen diversity (4, 28, 29). Moreover,
197 the coinfection model predicts that increased virulence is crucial when coinfected hosts are
198 common (3) because increased virulence can benefit pathogen transmission between hosts to
199 increase the exploitation rate (6). In keeping with these predictions, we observed decreased
200 diversity (Fig 3c) and increased virulence (Fig 4c) of horizontally transmitted pathogens over time.
201 Therefore, the virulence evolution and community dynamics of those non-seedborne pathogens
202 (non-SPs) reflect both the possible within-spot competition and the ecological feedback of the
203 increased host density of *A. adenophora* over time. In the invasion front, sporadically distributed
204 *A. adenophora* individuals encounter diverse native pathogens with low virulence due to
205 life-history trade-offs when infecting novel hosts (30); as more virulent strains evolve, they
206 exclude weakly virulent strains and, in turn, cause decreased pathogen diversity over time. This
207 process is similar to serial passage experiments (SPE), in which the initial infections with varied
208 mutants of virulence of one species are diverse, and virulence increases as the parasite is passaged
209 on the same host (31). Indeed, we observed a linear relationship between pathogen diversity and
210 virulence variation within spots (Fig 4a), which also implies that diverse pathogens with distinct
211 virulence likely coinfect hosts at early stages of invasion.
212 In contrast, vertically transmitted pathogens will lose virulence over time because their
213 fitness completely depends on hosts (3). The decreased virulence of the seedborne pathogens (SPs)
214 to the host *A. adenophora* over invasive time supported this idea (Fig 4c). Theoretically, most
215 models assuming that parasites are not transmitted vertically predict that parasites cannot increase
216 between-host transmission rates without increasing virulence, therefore weakly virulent pathogens
217 are competitively inferior in the case of mixed infections (3). In testing this prediction, however,
218 we observed that in older invasive areas, Didymellaceae (primary SPs) appeared to win by
219 increasing in abundance, as well as in occurrence frequency in leaf spots (Fig 1a, S7 Fig). Our
220 data indirectly suggest that vertically transmitted pathogens (SPs) can have a competitive
221 advantage over non-SPs due to the priority effects of within-host transmission (25); therefore the
222 evolution of low virulence may contrarily benefit the reproduction of SPs in the mixed infections
223 within leaf spots (32).
224 Escaping or encountering less virulent specialists if not escaping (33, 34) are important to
225 plant invasions. It is unclear if the decreased virulence of seedborne fungal pathogens directly
226 contribute to *A. adenophora* invasion because the foliar fungi as a whole in older sites produce
227 stronger adverse effects on the growth of *A. adenophora* than do those in younger sites (35). Here,
228 the evolution of decreased virulence of SPs may only be a response to the increased density of *A.*
229 *adenophora* over invasive history, as models predict that low-virulence strains prevail, and vertical
230 transmission becomes more prevalent, when host density increases if parasites are transmitted
231 both vertically and horizontally on a host (5). It appears that these SPs can also be horizontally
232 transmitted, as we found a high number of Didymellaceae spores in the canopy air of *A.*
233 *adenophora* (36).
234 A generalist strategy provides the parasite with more opportunities for transmission and
235 persistence; theoretically decreasing specificity can be associated with increasing virulence (37).
236 Coinfection also offers opportunities for hybridization among different species to enlarge host
237 range (38). However, over time no evolution on native plants, including host range and virulence,
238 occurred for both non-SPs and SPs (Fig 5b, c), although both types of pathogen evolved in
239 virulence to *A. adenophora* (Fig 4c), suggesting that the virulence to the host, for fungal pathogens
240 of leaf spots, is a more plastic trait than adaptation to novel hosts over time.
241 In conclusion, we employed an invasive plant as a model to answer the question of how
242 multiple infections of different fungal species with mixed vertical and horizontal transmission
243 mode affect virulence evolution. Over time, the horizontally transmitted pathogens evolved
244 increased virulence; in contrast, the vertically transmitted fungal pathogens evolved decreased
245 virulence. Meanwhile, all of these pathogens exhibited decreased species diversity (Fig 3c). This
246 study categorized vertically transmitted pathogens only using the sharing of seedborne fungi
247 without verifying their within-host growth systematically one by one (see Fig 2b). In fact, these
248 seedborne fungi (e.g., OTU1) can also horizontally transmit (see Chen, Fang (36)). In addition,
249 some non-SPs are expected to vertically transmit if dispersal of fragments of stems and rhizomes
250 by the flood occurs during the expansion of *A. adenophora* (21). Nonetheless, the invasive system
251 can be treated as a virulence evolution experiment in a realistic environment; indeed, many human,
252 animal and plant diseases exhibit mixed vertical and horizontal transmission (5) and simultaneous
253 infection by multiple species and genotypes of the same species (1, 2, 22, 23). Surprisingly, many
254 predictions in classical models regarding multiple infections of strains from the same species (3)
255 remain valid in such a complex system, in which the primary transmission mode (vertical vs
256 horizontal) determines the virulence evolution trajectory of pathogens in response to host density.
257 In addition, our finding that the assembly of foliar pathogens in leaf spots is related to
258 seedborne fungi may have far-reaching impacts on the understanding of what factors determine
259 foliar pathogen community structure. A complex set biotic and abiotic factors have
260 been thought to determine the foliar pathogen community (39); in particular, host plant
261 identification strongly affects the occurrence of foliar fungal pathogens (40, 41). Until very
262 recently, the primary symbiont hypothesis (PSH) proposed that a single genotype (the seedborne
263 primary symbiont) has strong effects on the microbiome assembly of seedlings (25). Here we
264 propose that it is important to identify whether the primary foliar pathogen of a given plant host is
265 relevant to seedborne fungi, which play important roles in structuring the pathogen community of
266 multiple infections (mostly horizontally transmitted from the surroundings). In fact, the infection
267 of a primary foliar pathogen may be common in native (42) and invasive plants (11), but it
268 remains to be verified whether this pattern is relevant to seedborne fungi. On this point, we also
269 emphasize studying pathogens at the genotype level (43) because identifying a given genotype,
270 rather than one species, may be crucial to elucidating the pathogen community of multiple
271 infections.

## 272 4. Materials and Methods

### 273 4.1 Study sites and sample collections

274 Previously, we reported the foliar pathogen sharing of *A. adenophora* with local plants in
275 Yunnan Province, which is a relatively old invasion area (20). In this case, we expanded the
276 sampling areas into two newly established neighbouring provinces, Guizhou and Guangxi.
277 Because we were concerned with multiple within-leaf spot infections, previous pathogens without
278 clear leaf spot sources were excluded from the analysis. According to the geographical expansion
279 dynamics of *A. adenophora* in Southwest China (18), a total 14 sites belonging to 80-, 50- and
280 20-year-old areas were selected for investigation of leaf spots. At each site, leaf spots with
281 different symptoms were collected from *A. adenophora* and totally 236 leaf spots were subject to
282 isolate leaf spot fungi within 12 hours after collection (Table 1, S1 File).
283 **Table 1** Geographical information, population distribution and number of leaf spots in three
284 invasive times.

| **Invasive time ^a^** | **Sample sites** | **Province** | **Longitude (°)** | **Latitude (°)** | **Elevation (m)** | **No. of leaf spots** |
| --- | --- | --- | --- | --- | --- | --- |
|  | CY | Yunnan | 99.31 | 23.22 | 1708 | 25 |
| 80 years | LC  SM | Yunnan  Yunnan | 99.80  100.81 | 22.63  22.75 | 1715  1171 | 38  22 |
|  | NE | Yunnan | 101.05 | 23.00 | 1391 | 21 |
|  | XS | Yunnan | 102.62 | 24.98 | 2080 | 25 |
| 50 years | YX  NJ | Yunnan  Yunnan | 100.03  100.30 | 24.28  24.87 | 1340  1730 | 24  20 |
|  | ES | Yunnan | 102.28 | 24.12 | 1748 | 17 |
|  | NY | Guizhou | 105.58 | 26.83 | 1160 | 5 |
|  | PT | Guizhou | 107.06 | 25.84 | 660 | 9 |
| 20 years | DS  ND | Guizhou  Guangxi | 107.52  107.51 | 25.36  25.03 | 900  800 | 8  7 |
|  | TL | Guangxi | 106.23 | 24.29 | 340 | 7 |
|  | DB | Guangxi | 106.57 | 23.34 | 700 | 8 |

285 **Note:** ^a^ Invasive time for each site is referred to (18). CY represents Cangyuan county, LC represents Lancang
286 county, SM represents Simao city, NE represents Ninger county, XS represents Xishan district in Kunming city,
287 YX represents Yunxian county, NJ represents Nanjian county, ES represents Eshan county, NY represents Nayong
288 county, PT represents Pingtang county, DS represents Dushan county, ND represents Nandan county, TL represents
289 Tianlin county, DB represents Debao county.

### 290 4.2 Isolation and identification of leaf spot fungi

291 We focused on isolating necrotrophic pathogens because symptoms have rarely been
292 observed on the leaves of *A. adenophora* for any of the biotrophic pathogens. The leaves were
293 rinsed with tap water and then surface sterilized (2% sodium hypochlorite for 30 s	and 75%
294 ethanol for 2 min and rinsed with sterile water 3 times). Healthy leaf tissues and the margins of
295 diseased tissues of each leaf spot were cut into twelve ~6-mm^2^ sections. For leaf spots with the
296 size< 2 mm in diameter which is unable to cut into 12 fragments, and 3-4 leaf spots with the same
297 symptoms from one leaf were used to represent one leaf spot. The disinfected fragments were
298 subsequently plated onto potato dextrose agar (PDA) and incubated at ambient temperature for
299 6–8 d. All fungal colonies grown from the leaf fragments were purified and used in phylogenetic
300 analysis and disease experiments. The fungi were maintained as pure cultures at Yunnan
301 University (Kunming, China).
302 Total genomic DNA was extracted from fungal mycelia using the CTAB method (44). The
303 internal transcribed spacer (ITS) region was amplified with the fungal primer pair ITS4 and ITS5
304 and PCR products were sent to Sangon Biotech Co., Ltd. (Shanghai, China) for ITS sequencing.
305 Based on the GenBank and UNITE databases, sequence homology analysis, quality assessment
306 and correction were conducted. ClustalX 2.1 was used to cut out chimeric bases to make each
307 sequence measure approximately 550 bp in length (45).
308 We grouped fungi into operational taxonomic units (OTUs) based on ITS locus because
309 using traditional morphological species recognition and biological species recognition of fungi is
310 operationally difficult due to the unavailability of taxonomically informative morphologies or
311 mating systems for many of the cultured fungi (46). We analysed the fungal community using
312 OTUs based on 100% cutoffs, which overlooks the intraspecific and intragenomic variations
313 among ITS copies in fungi (47). MOTHUR 1.35.1 with the Naïve Bayesian classifier was used to
314 identify the remaining sequences (database, UNITE_public_mothur_full_04.02.2020 (48)) and to
315 group the consensus sequences into OTUs (49). Because different trimming of alignment and the
316 updated version of UNITE database were used in this analysis, which caused changes partially in
317 the delimitation and phylogeny of OTUs that previously reported (20), these ITS sequences were
318 resubmitted and all nucleotide sequences reported in this study were deposited at GenBank under
319 the accession numbers MT908377-MT908827(also see S1 File).

### 320 4.3 Detection of virulence and host range of leaf spot fungi

321 We used foliar damage as a virulence evaluation in this study. We first tested the virulence of
322 all isolated fungi on host *A. adenophora*. The inoculation experiment was performed as previously
323 reported to test the virulence of necrotrophic pathogens in tropical forests (50) with minor
324 modifications, as detailed in a previous report (20). Each fungal strain was inoculated into five
325 mature and healthy leaves from a single individual of *A. adenophora* to test pathogenicity. At 1
326 week after inoculation, leaves were harvested and photographed, and lesions were
327 measured. The disease experiment was performed over multiple years in the field, and
328 environmental factors, such as moisture and temperature, were not controlled. However, all
329 inoculation experiments were performed between June and the end of October, the primary growth
330 season for plants in Kunming. Then, to further determine the virulence to native plants and host
331 range of the verified pathogens of *A. adenophora*, fungal pathogens of *A. adenophora* were
332 randomly selected to perform the pathogenicity test on nine native plants.
333 **4.4 Investigation of seedborne fungi of *A. adenophora***
334 Because the vertical transmission of fungi in most plant seeds is imperfect and isolation
335 frequency is commonly low (25), we performed high-throughput sequencing using bulked seed
336 samples, rather than individual seeds. High-throughput sequencing was performed on seeds
337 collected from 9 populations of *A. adenophora* in Yunnan Province (S1 File). To identify the
338 fungi shared in foliar and seed samples of *A. adenophora*, the representative ITS sequences
339 from two libraries were selected and aligned. The ITS sequence obtained by the
340 high-throughput sequencing technology was short (~250 bp), and the alignment was trimmed to
341 this range to cluster into OTUs using MOTHUR software with a 100% similarity cutoff (51).
342 This trimming	caused	several OTUs to merge	in	both	libraries. Deletions/insertions were
343 Included when comparing sequences. The OTUs that were identical from both sources were defined
344 as shared fungi. The shared leaf spot pathogens were defined as seedborne pathogens (SPs); in
345 contrast, leaf spot fungi with no exact matches to seedborne fungi were defined as non-seedborne pathogens (non-SPs). The
346 next-gen data were submitted to GenBank under bioproject accession numbers PRJNA657950
347 (also see S1 File).

### 348 4.5 Data analysis

349 At the macroscale level (geographic site), we pooled all fungi from each geographic site as an
350 independent sampling to calculate and compare the pathogen diversity. At the microscale level
351 (leaf spot), only the leaf spots with multiple infections were analysed, the pathogens in each leaf
352 spot were treated as a pathogen community (an independent sampling), and pathogen diversity
353 was calculated and compared; meanwhile, only those pathogens in leaf spots with multiple
354 infections were used to evaluate virulence evolution and pathogen dynamics over time based on
355 the categories of vertical and horizontal transmission mode.
356 Nonparametric analysis with the Mann-Whitney U test was performed using SPSS v.22.0
357 software (SPSS Inc., Chicago, IL, USA) to compare the differences of all parameters between
358 invasive times, including Shannon diversity, average virulence and native host range of fungal
359 pathogens. Principal coordinate analysis (PCoA) visualized the similarity in fungal composition,
360 and redundancy analysis (RDA) tested the correlations between fungal communities and invasion
361 time using CANOCO v.5.0 software (52). All data represented fungal abundance, and distance
362 matrices were constructed based on the Bray-Curtis dissimilarity index. The maximum likelihood
363 estimation was performed in 1000 replicates through rapid bootstrap analysis in MEGA 7.0 to
364 show the phylogenetic clustering of strains (53). The coefficient of variation (CV) was calculated
365 by dividing the standard deviation by the mean to represent the virulence variation of fungal
366 pathogens. Pearson's coefficient matrix was calculated using R package “ggally” (54) to analyse
367 the correlation between the virulence of fungal pathogens to *A. adenophora*, the virulence to
368 native species and the host range on native species. Linear regression was employed to analyse the
369 relationship between virulence variation and pathogen diversity (SPSS v.22.0).
370 A circular barplot was used to show the proportion of the fungal pathogen *A. adenophora* at
371 three invasion times (R package “ggplot2” (55)). The arc diagram shows the shared OTUs
372 between leaf spots and seeds (R package “ggraph” (56)). Boxplots or barplots of Shannon
373 diversity, average virulence and virulence variation of *A. adenophora* pathogens were performed
374 using GraphPad Prism 7 (GraphPad Software Inc., San Diego, CA, USA). A pie chart was used to
375 show the species richness on each leaf spot, and a doughnut was used to show the occurrence
376 frequency and relative abundance of SPs and non-SPs (GraphPad Prism 7). Density curves and
377 bubble plots were utilized to show the virulence distribution of fungal pathogens to *A. adenophora*
378 (R package “ggplot2” (55)). A correlogram was drawn to visualize the scatter matrix and density
379 distribution (R package “ggplot2” (55)).

## 381 Data accessibility

382 The nucleotide sequences reported in this study have been deposited in GenBank under
383 accession numbers MT908377-MT908827. The next-gen data were submitted to GenBank under
384 bioproject accession numbers PRJNA657950. Data supporting the results are attached to
385 Supplementary Information S1 File.

## 387 Author Contributions

388 **Conceptualization:** D. Han-Bo Zhang.
389 **Data Curation:** Kai Fang, Jie Zhou.
390 **Formal Analysis:** Kai Fang, Jie Zhou, D. Han-Bo Zhang.
391 **Funding Acquisition:** D. Han-Bo Zhang.
392 **Investigation:** Kai Fang, Jie Zhou, Lin Chen, Yu-Xuan Li, Ai-Ling Yang, Xing-Fan Dong, D.
393 Han-Bo Zhang.
394 **Methodology:** Kai Fang, Jie Zhou, Lin Chen, Yu-Xuan Li, Ai-Ling Yang, Xing-Fan Dong, D.
395 Han-Bo Zhang.
396 **Project Administration:** D. Han-Bo Zhang.
397 **Resources:** Kai Fang, Jie Zhou, Lin Chen, Yu-Xuan Li, Ai-Ling Yang, Xing-Fan Dong, D.
398 Han-Bo Zhang.
399 **Software:** Kai Fang.
400 **Supervision:** D. Han-Bo Zhang.
401 **Validation:** Jie Zhou.
402 **Visualization:** Kai Fang.
403 **Writing – Original Draft Preparation:** Kai Fang, Jie Zhou.
404 **Writing – Review & Editing:** D. Han-Bo Zhang.

## 406 Competing interests

407 The authors declare that they have no conflicts of interest.

## 409 Funding

410 This work was supported by the National Natural Science Foundation of China (grant nos.

411	31770585 and 31360153).

412

## 413 Acknowledgements

414 The authors thank Tian Zeng, Li-Yuan Qin, Wen-Ti Zheng, and Zhi-Ping Yang at Yunnan
415 University for help with sampling in the field and performing the disease experiment. Dr
416 Huan-Chong Wang and Dr Tao Xu at Yunnan University assisted with the identification of plant
417 species.

## 419 References

420 1.	Lamichhane JR, Venturi V. Synergisms between microbial pathogens in plant disease complexes:
421 a growing trend. Front Plant Sci. 2015;6:385.
422 2.	Tollenaere C, Susi H, Laine A-L. Evolutionary and epidemiological implications of multiple
423 infection in plants. Trends Plant Sci. 2016;21(1):80-90.
424 3.	Alizon S, de Roode JC, Michalakis Y. Multiple infections and the evolution of virulence. Ecol

425	Lett. 2013;16(4):556-67.

426 4.	Vanbaalen M, Sabelis MW. The scope for virulence management: a comment on Ewald's view on
427 the evolution of virulence Trends Microbiol. 1995;3(11):414-6.
428 5.	Lipsitch M, Siller S, Nowak MA. The evolution of virulence in pathogens with vertical and
429 horizontal transmission. Evolution. 1996;50(5):1729-41.
430 6.	Hood ME. Dynamics of multiple infection and within-host competition by the anther-smut

431	pathogen. Am Nat. 2003;162(1):122-33.

432	7.	Callaway RM, Maron JL. What have exotic plant invasions taught us over the past 20 years?

433	Trends Ecol Evol. 2006;21(7):369-74.

434	8.	Lambrinos JG. How interactions between ecology and evolution influence contemporary invasion

435	dynamics. Ecology. 2004;85(8):2061-70.

436	9.	Keane RM, Crawley MJ. Exotic plant invasions and the enemy release hypothesis. Trends Ecol

437	Evol. 2002;17(4):164-70.

438	10. Flory SL, Clay K. Pathogen accumulation and long-term dynamics of plant invasions. J Ecol.

439	2013;101(3):607-13.

440	11. Stricker KB, Harmon PF, Goss EM, Clay K, Flory SL. Emergence and accumulation of novel

441	pathogens suppress aninvasive species. Ecol Lett. 2016;19(4):469-77.

442	12. Mitchell CE, Blumenthal D, Jarosik V, Puckett EE, Pysek P. Controls on pathogen species

443	richness in plants' introduced and native ranges: roles of residence time, range size and host traits. Ecol

444	Lett. 2010;13(12):1525-35.

445	13. Clay K. Correlates of pathogen species richness in the grass family Can J Bot. 1995;73(S1):42-9.

446	14. Garrett KA, Mundt CC. Epidemiology in mixed host populations. Phyto. 1999;89(11):984-90.

447	15. Bufford JL, Hulme PE, Sikes BA, Cooper JA, Johnston PR, Duncan RP. Taxonomic similarity,

448	more than contact opportunity, explains novel plant-pathogen associations between native and alien

449	taxa. New Phytol. 2016;212(3):657-67.

450	16. Dickie IA, Bufford JL, Cobb RC, Desprez-Loustau M-L, Grelet G, Hulme PE, et al. The emerging

451	science of linked plant-fungal invasions. New Phytol. 2017;215(4):1314-32.

452	17. Poudel AS, Jha PK, Shrestha BB, Muniappan R. Biology and management of the invasive weed

453	*Ageratina adenophora* (Asteraceae): current state of knowledge and future research needs. Weed Res.

454	2019;59(2):79-92.

455	18. Wang R, Wang Y-Z. Invasion dynamics and potential spread of the invasive alien plant species

456	*Ageratina adenophora* (Asteraceae) in China. Diversity Distrib. 2006;12(4):397-408.

457	19. Zhou Z-X, Jiang H, Yang C, Yang M-Z, Zhang H-B. Microbial community on healthy and

458	diseased leaves of an invasive plant *Eupatorium adenophorum* in Southwest China. J Microbiol.

459	2010;48(2):139-45.

460	20. Chen L, Zhou J, Zeng T, Miao YF, Mei L, Yao GB, et al. Quantifying the sharing of foliar fungal

461	pathogens	by	the	invasive	plant	*Ageratina	adenophora*	and	its	neighbours. New Phytol.

462	2020;227:1493-504.

463 21. Wang R, Wang J-F, Qiu Z-J, Meng B, Wan F-H, Wang Y-Z. Multiple mechanisms underlie rapid
464 expansion of an invasive alien plant. New Phytol. 2011;191(3):828-39.
465 22. Crous PW, Groenewald JZ, Summerell BA, Wingfield BD, Wingfield MJ. Co-occurring species of
466 *Teratosphaeria* on *Eucalyptus*. Persoonia. 2009;22:38-48.
467 23. Perez G, Slippers B, Wingfield BD, Hunter GC, Wingfield MJ. Micro- and macrospatial scale
468 analyses illustrates mixed mating strategies and extensive geneflow in populations of an invasive
469 haploid pathogen. Mol Ecol. 2010;19(9):1801-13.
470 24. Bass D, Stentiford GD, Wang H-C, Koskella B, Tyler CR. The Pathobiome in Animal and Plant

471	Diseases. Trends Ecol Evol. 2019;34(11):996-1008.

472	25. Newcombe G, Harding A, Ridout M, Busby PE. A Hypothetical Bottleneck in the Plant

473	Microbiome. Front Microbiol. 2018;9:1645.

474	26. Jiang H, Shi Y-T, Zhou Z-X, Yang C, Chen Y-J, Chen L-M, et al. Leaf chemistry and co-occurring

475	species interactions affecting the endophytic fungal composition of Eupatorium adenophorum. Ann

476	Microbiol. 2011;61:655-62.

477	27. Fang K, Miao Y-F, Chen L, Zhou J, Yang Z-P, Dong X-F, et al. Tissue-specific and geographical

478	variation in endophytic fungi of *Ageratina adenophora* and fungal associations with the environment.

479	Front Microbiol. 2019;10.

480	28. May RM, Anderson RM. Epidemiology and genetics in the coevolution of parasites and hosts.

481	Proc R Soc B. 1983;219(1216):281-313.

482	29. Nowak MA, May RM. Superinfection and the evolution of parasite virulence. Proc R Soc B.

483	1994;255(1342):81-9.

484 30. Laine AL, Barres B. Epidemiological and evolutionary consequences of life-history trade-offs in
485 pathogens. Plant Pathol. 2013;62:96-105.
486 31. Ebert D. Experimental evolution of parasites. Science. 1998;282(5393):1432-5.
487 32. Choisy M, de Roode JC. Mixed infections and the evolution of virulence: effects of resource
488 competition, parasite plasticity, and impaired host immunity. Can J Bot. 2010;175(5):E105-E18.
489 33. Reinhart KO, Tytgat T, Van der Putten WH, Clay K. Virulence of soil-borne pathogens and
490 invasion by *Prunus serotina*. New Phytol. 2010;186(2):484-95.
491 34. Vandegrift R, Blaser W, Campos-Cerda F, Heneghan AF, Carroll GC, Roy BA. Mixed fitness
492 effects of grass endophytes modulate impact of enemy release and rapid evolution in an invasive grass.

493	Biol Invasions. 2015;17(4):1239-51.

494	35. Fang K, Chen L, Zhou J, Yang ZP, Dong XF, Zhang HB. Plant-soil-foliage feedbacks on seed

495	germination and	seedling growth of the	invasive	plant	*Ageratina adenophora*. Proc	R Soc B.

496	2019;286(1917):20191520.

497 36. Chen L, Fang K, Dong XF, Yang AL, Li YX, Zhang HB. Characterization of the fungal
498 community in the canopy air of the invasive plant *Ageratina adenophora* and its potential to cause
499 plant diseases. PLoS One. 2020;15(3):e0230822.
500 37. Barrett L, Kniskern J, Bodenhausen N, Zhang W, Bergelson J. Continua of specificity and
501 virulence in plant host-pathogen interactions: causes and consequences. New Phytol. 2009;183:513-29.
502 38. Inderbitzin P, Davis R, Bostock R, Subbarao K. The Ascomycete *Verticillium longisporum* is a
503 hybrid and a plant pathogen with an expanded host range. PloS one. 2011;6:e18260.
504 39. Blakeman J. Pathogens in the foliar environment. Plant Pathol. 2007;42:479-93.
505 40. Hönig L, Braun U, Scherer-Lorenzen M, Bruelheide H. Species richness and species identity
506 effects on occurrence of foliar fungal pathogens in a tree diversity experiment. Ecosphere.

507	2013;4:art81.

508	41. Hantsch L, Braun U, Haase J, Purschke O, Scherer-Lorenzen M, Bruelheide H. No plant

509	functional diversity effects on foliar fungal pathogens in experimental tree communities. Fungal Divers.

510	2014;66(1):139-51.

511	42. Garcia-Guzman G, Dirzo R. Patterns of leaf-pathogen infection in the understory of a Mexican

512	rain forest: Incidence, spatiotemporal variation, and mechanisms of infection. Am J Bot.

513	2001;88(4):634-45.

514 43. Callahan BJ, McMurdie PJ, Rosen MJ, Han AW, Johnson AJA, Holmes SP. DADA2:
515 High-resolution sample inference from Illumina amplicon data. Nat Methods. 2016;13(7):581-3.
516 44. Stewart CN, Via L. A rapid CTAB DNA isolation technique useful for RAPD fingerprinting and
517 other PCR amplifications. BioTechniques. 1993;14:748-50.
518 45. Thompson JD, Gibson TJ, Plewniak F, Jeanmougin F, Higgins DG. The CLUSTAL_X windows
519 interface: flexible strategies for multiple sequence alignment aided by quality analysis tools. Nucleic

520	Acids Res. 1997;25(24):4876-82.

521	46. Gazis R, Rehner S, Chaverri P. Species delimitation in fungal endophyte diversity studies and its

522	implications in ecological and biogeographic inferences. Mol Ecol. 2011;20(14):3001-13.

523	47. Simon UK, Weiss M. Intragenomic variation of fungal ribosomal genes is higher than previously

524	thought. Mol Biol Evol. 2008;25(11):2251-4.

525 48. Abarenkov K, Zirk A, Piirmann T, Pöhönen R, Ivanov F, Nilsson RH, et al. Urmas (2020): Full
526 mothur UNITE+INSD dataset 2. UNITE Community. 2020.
527 49. Schloss PD, Westcott SL, Ryabin T, Hall JR, Hartmann M, Hollister EB, et al. Introducing mothur:
528 open-source, platform-independent, community-supported software for describing and comparing
529 microbial communities. Appl Environ Microbiol. 2009;75(23):7537-41.
530 50. Gilbert GS, Webb CO. Phylogenetic signal in plant pathogen–host range. Proc Natl Acad Sci.

531	2007;104(12):4979-83.

532	51. Edgar RC. UPARSE: highly accurate OTU sequences from microbial amplicon reads. Nat

533	Methods. 2013;10(10):996-+.

534 52. Šmilauer P, Lepš J. Multivariate analysis of ecological data using CANOCO 5, 2nd edn. New
535 York, NY: Cambridge University Press. 2014.
536 53. Kumar S, Stecher G, Tamura K. MEGA7: molecular evolutionary genetics analysis version 7.0 for
537 bigger datasets. Mol Biol Evol. 2016;33(7):1870-4.
538 54. Schloerke B, Crowley J, Cook D, Hofmann H, Wickham H, Briatte F, et al. GGally: Extension to
539 ggplot. R package version 0.5.0. Available from: [http://CRANR-projectorg/package/GGally.](http://cranr-projectorg/package/GGally) 2014.
540 55. Ginestet C. ggplot2: Elegant Graphics for Data Analysis. J R Stat Soc A Stat. 2011;174:245-.
541 56. Pedersen TL. ggraph: An Implementation of Grammar of Graphics for Graphs and Networks
542 Available from: [https://CRANR-projectorg/package=ggraph](https://cranr-projectorg/package%3Dggraph) 2020.

## 545 Figure legend

546 **Fig. 1 Isolation and virulence verification of leaf spot fungi.** (a) The proportion of *A.*
547 *adenophora* pathogens at the family (left) and OTU levels (right). Families with relative
548 abundance > 2% are shown, and the rest were merged into "Others"; OTUs with relative
549 abundance > 1% are shown. (b) Leaf spots with varying fungal species of pathogens. The number
550 in each section represents the number of leaf spots with different pathogen richness. The green
551 sections are leaf spots with multiple infections, and the grey section is leaf spot with a single
552 infection.
554 **Fig. 2 Shared OTUs between leaf spot fungi and seedborne fungi.** (a) The Venn diagram shows
555 the unique and shared OTU number (outside brackets) and abundance (reads) (inside brackets)
556 between libraries of leaf spots and seeds. (b) The network diagram detailed the 17 shared OTUs
557 between the leaf spots and the seeds. Didy, Didymellaceae; Xyle, Xylariales; Nect, Nectriaceae;
558 Pleo, Pleosporaceae; Cera, Ceratobasidiaceae. The numbers in brackets represent abundance for
559 leaf spot isolates and for seed high-throughput sequencing reads, respectively. Because the ITS
560 sequence obtained by the high-throughput sequencing technology was short (~250 bp), the
561 alignment was trimmed to this range and was clustered to generate OTUs again. This trimming
562 caused a few OTUs to merge in both libraries (see Methods).

### 564 Fig. 3 Diversity and structure of fungal pathogens over invasive history at leaf spots with

565 **multiple infections.** Diversity (a) and structure (b) of fungal pathogens at the leaf spot level at
566 different invasion times. (c) Diversity of seedborne pathogens (SPs) and non-seedborne pathogens
567 (non-SPs) at the leaf spot level at different invasion times. Each point represents one leaf spot with
568 multiple infections. In panels (a) and (c), nonparametric analysis with the Mann-Whitney U test
569 was performed to show that the Shannon diversity index difference was significant among
570 different invasion times by different lowercase letters (*p* < 0.05). In panel (b), principal coordinate
571 analysis (PCoA) showed the similarity of pathogenic communities among leaf spots with multiple
572 infections. SP represents leaf spot containing SP but may also cooccur with non-SPS; non-SP
573 represents leaf spot containing only non-SPs. Percentages of total explained variation by PCoA
574 axes in each plot are given in parentheses. (d) The occurrence frequency and relative abundance in
575 leaf spots of SP and non-SP at different invasion times.
577 **Fig. 4 Fungal virulence evolution on host *A. adenophora.*** (a) Linear analysis of within-spot
578 species diversity and virulence variations. Each spot represents a leaf spot with multiple infections.
579 (b) The density plots show the virulence distribution of fungal pathogens to *A. adenophora* at
580 different invasion times. The boxplots show the differences in the average virulence (up) and the
581 virulence variation (below) between different invasion times. (c) Virulence variation within spots
582 of SP and non-SP at different invasion times. Nonparametric analysis with the Mann-Whitney U
583 test was performed to test the significance of differences, and different lowercase letters indicate
584 significant differences (*p* < 0.05). Differences between SP and non-SP of each invasion time were
585 also given. The leaf spot area (mm^2^) is shown as square root transformed. The error bar in panel (c)
586 represents the standard deviation.
588 **Fig. 5 The virulence and host range of SP and non-SP fungi.** (a) Virulence difference either of
589 SP or of non-SP fungi to *A. adenophora* and native plants. (b) Virulence difference and (c) host
590 range between SP and non-SP fungi to native plants. Nonparametric analysis with the
591 Mann-Whitney U test was performed to test the significance of differences, and different
592 lowercase letters indicate significant differences (*p* < 0.05). Differences between SP and non-SP of
593 each invasion time were also given. The error bar in panel (a) represents the standard deviation.

## 595 Supporting information

### 596 S1 Fig. Relative abundance of numerically dominant fungi at family level. (a) Total isolated

597 fungi. (b) The verified pathogenic fungi to *A. adenophora*.
599 **S2 Fig. Rarefaction curves of high-through sequencing in seeds.** The geographic information
600 for *A. adenophora* population detailed in S1 File.

### 602 S3 Fig. Diversity (a) and structure (b) of fungal pathogens at geographic site level.

603 Nonparametric analysis with Mann-Whitney U test was performed to show that Shannon diversity
604 index difference significant among invasive times by different lowercase letters (*p* < 0.05).
605 Principal co-ordinates analysis (PCoA) showed the similarity of pathogenic fungi communities
606 among *A. adenophora* populations. Each spot represents one geographic site (detail see Table S1).
607 Percentages of total explained variation by PCoA axes in each plot are given in parentheses.

### 609 S4 Fig. Virulence distribution of total fungal pathogens to *A. adenophora*.

611 **S5 Fig. Abundance of fungal pathogens with different virulence to *A. adenophora* at family**
612 **level (a) and OTU level (b).** Families with relative abundance greater than 1% are shown, and the
613 14 relatively abundant family accounted for 86.2% of the total. OTUs with relatively abundance
614 greater than 1% are shown, and the 19 relatively abundant OTUs accounted for 50.1% of the total.
615 The families (or OTUs) were clustered according to phylogenetic position, and the posterior
616 probability are indicated at the branch node. According to the density distribution of virulence of
617 fungal pathogens to *A. Adenophora*, 5 virulence ranges (LSA ≤ 50 mm^2^, 50 < LSA ≤ 100 mm^2^,

618	100 < LSA ≤ 150 mm^2^, 150 < LSA ≤ 200 mm^2^, 200 < LSA ≤ 500 mm2 and LSA > 500 mm^2^) were

619	selected to display the data.

620

621 **S6 Fig. The host range and virulence of fungi to native plants.** (a) Host range. The number
622 above each bar represents the number of isolates. (b) Pearson correlations among native host
623 number, virulence to *A. Adenophora* and average virulence to native plants of fungal pathogens. (c)
624 Average host range and average virulence to native plants of 184 selected fungi over invasive time.
625 Nonparametric analysis with Mann-Whitney U test was performed to test the significant of
626 differences, and different lowercase letters indicate significant differences (*p* < 0.05). Error bar
627 represents standard error.

### 629 S7 Fig. Occurrence frequency and relative abundance of SP and nonSP in leaf spots with

630 **single infection in different invasion time.**

### 632 S1 File. Raw data used in this study included the leaf spot disease incidence , cultivable fungi

633 **of leaf spot, high-throughput fungal sequencing of seeds and native host data.**

Figure	[Click here to access/download;Figure;Figure 1.tif](https://www.editorialmanager.com/ppathogens/download.aspx?id=1547696&guid=4d3ddca6-2e69-4f4c-9491-4b1c76721cc6&scheme=1)

Figure	[Click here to access/download;Figure;Figure 2.tif](https://www.editorialmanager.com/ppathogens/download.aspx?id=1547697&guid=8c332543-8c1a-4a59-bd70-a14d6b6af5e4&scheme=1)

Figure	[Click here to access/download;Figure;Figure 3.tif](https://www.editorialmanager.com/ppathogens/download.aspx?id=1547699&guid=a58f5253-3b3b-465e-8dca-d105ead635c3&scheme=1)

Figure	[Click here to access/download;Figure;Figure 4.tif](https://www.editorialmanager.com/ppathogens/download.aspx?id=1547702&guid=37607dd5-923d-4160-8911-32648c8a8de1&scheme=1)

Figure	[Click here to access/download;Figure;Figure 5.tif](https://www.editorialmanager.com/ppathogens/download.aspx?id=1547703&guid=387e559c-e107-4e29-a252-2bd2e57b1d75&scheme=1)

Supporting Information

[Click here to access/download](https://www.editorialmanager.com/ppathogens/download.aspx?id=1547716&guid=098a1d58-0356-4dc6-87c6-b43ec05843ae&scheme=1)

# [Supporting Information Supplementary materials.pdf](https://www.editorialmanager.com/ppathogens/download.aspx?id=1547716&guid=098a1d58-0356-4dc6-87c6-b43ec05843ae&scheme=1)

Supporting Information

[Click here to access/download](https://www.editorialmanager.com/ppathogens/download.aspx?id=1547717&guid=b11054d5-176c-4700-b649-50f969b637fc&scheme=1)

# [Supporting Information S1 File.xlsx](https://www.editorialmanager.com/ppathogens/download.aspx?id=1547717&guid=b11054d5-176c-4700-b649-50f969b637fc&scheme=1)
