# Supplementary material for: Virulence and community dynamics of fungal species with vertical and horizontal transmission on a plant with multiple infections
Source: PLoS Pathog. 2021 Jul 15;17(7):e1009769. doi: 10.1371/journal.ppat.1009769 (PMC8315517; doi:10.1371/journal.ppat.1009769)
Supplement: Attachment — Submitted filename: Response to reviewers.docx [file ppat.1009769.s011.docx]

Dear reviewers:

We greatly appreciate your efforts to improve the quality of our manuscript. We have agreed to include all the changes proposed by you and to revise the manuscript for clarity and readability. To facilitate the review process, brief point-by-point responses to the queries or comments made by you are provided below. Your comments are shown in black, and our responses in blue. Thank you very much.

**Part I – Summary**

Reviewer #1:

Here the authors evaluate a theoretical evolutionary model where vertically transmitted pathogens reduce in virulence whereas horizontally transmitted pathogens increase in virulence in association with an invasive host species proving that classical prediction models are valid in a complex natural environment involving an invasive plant species.
The work done here is interesting in that it uses a naturally occurring invasion of the plant species *Ageratina adenophora* and its vertically and horizontally transmitted foliar fungal pathogens to set up a model to test currently held evolutionary theories. Good techniques and data analysis appear to have been used and the conclusions are justified, however, I am not convinced that the outcomes of this study are novel enough or of broad enough interest to be published in PLoS Pathogens.

Response: Thank you very much.

Reviewer #2:

This paper describes results from a study of an invasive plant *Ageratina adenophora* in three sites where it was introduced 20, 50 and 80 years ago, and the community structure of its foliar pathogens. Using a combination of methods, the authors found that pathogen diversity decreased over time, while vertically and horizontally transmitted pathogens showed decreased and increased virulence, respectively. This is consistent with predictions of evolutionary models.
This is a very nice system and the authors used a variety of methods to characterize it. The data in this paper represent quite a lot of work. I like that the way that they have applied this model system to demonstrate a pattern that was predicted by evolutionary theory. This system shows promise for studies of invasive plant control, as well as plant disease dynamics in general.

Response: Thank you very much.

Reviewer #3:

This paper reports on changing virulence profiles in several fungal pathogens of an invasive plant *Ageratina adenophora* across several sites. The study aims to test the hypothesis that the trajectory of virulence evolution differs among horizontally and vertically transmitted pathogens. The authors find that pathogen virulence seems to evolve to higher levels among horizontally transmitted fungal OTUs while attenuates in vertically transmitted (or, more accurately, fungi found in both seeds and leaf spots). On the whole I think the results are suggestive and informative.
I am hard pressed to characterize the results as an "experimental evolution" study. Such studies begin with the ancestral genotype and through controlled conditions experimentally evolve organisms over several generations. The authors claim that the invasion process provides such a "natural experiment" but neither I nor any one else involved in experimental evolution will be much convinced this description is really an experimental test of how the mode of transmission affects virulence evolution. If the authors inoculated these plants over multiple generations in a green house with different kinds of pathogens and compared how the pathogens evolved during the course of that evolutionary experiment that would be one thing. This is fundamentally a different kind of test.
This is at heart an observational study using comparisons of different field populations. I think such studies can be immensely powerful and can help interrogate evolutionary theory in interesting ways, but their limitations are very well known. But the authors for instance cannot control for microhabitat differences, different host pools and other potential selective factors across the sites in Table 1. I think the use of a large number of OTUs helps mitigate this issue, but these constraints on occasion could be better emphasized.

Response: Thank you very much. We agreed your comments on this point. Our research is not a real experimental evolution study, which commonly performs in a control manner in greenhouse. However, unlike those evolution experiments for a single infection (e.g., single species, or single genotype of pathogen), an evolution experiment for a complex infection system, i.e., multiple infections involving multiple species or multiple genotypes of pathogens, combination with vertical and horizontal transmission modes, is difficult to perform in a greenhouse by artificial inoculation on a succession of generations. In particular, one very realistic difficult is to determine which pathogen is inoculated first and then which one is following…, because the first arrival of pathogen will greatly impact the colonization of following pathogens and thus change the evolutionary track; secondly, it is also difficult to obtain seeds infected with a specific fungi by growing a plant in greenhouse because the vertical transmission of fungi in most plant seeds is imperfect and isolation frequency is commonly very low (e.g., only 16 isolates are obtained from 800 seeds of *Pinus monticola* with one isolate per seed, see Newcombe G, Harding A, Ridout M, Busby PE. A Hypothetical Bottleneck in the Plant Microbiome. Front Microbiol. 2018; 9: 1645.). Therefore, an observational study occurring in the nature may be a good alternative to a control experiment in current stage.

Considering the limitations of our system, as you pointed, to be avoid of readers’ misunderstanding that our study is an evolutionary experiment in a control condition, i.e., serial passage of starting strains with varied distinct virulence, we made a substantial reversion to clarify this point, including: **(i)** title: changed as “Virulence and community dynamics of fungal species with vertical and horizontal transmission on a plant with multiple infections” **(lines 3-4)**; **(ii)** In the discussion section, we added sentences as: “Classical theory predicts that virulence evolution in multiple infections depends on the pathogen transmission mode and population dynamics of the pathogen, as well as on the host; however, for plant diseases, there is still a lack of experimental studies (3). Regarding the great challenge in performing serial passages in the laboratory or greenhouse by inoculating multiple different species with distinct levels of virulence, as well as with vertical and horizontal transmission on a host, we attempted to **use the epidemiological investigation** of *A. adenophora* and its foliar pathogens, a naturally occurring plant host–parasite system, as an alternative way to verify theoretical predictions.” **(lines 210-217);** **(iii)** In the conclusion paragraph, we added some sentences to discuss our limitation: “However, our data were mainly based on epidemiological investigations and comparisons of different field populations of *A. adenophora*, without considering microhabitat differences or other potential selective factors across the sites. In addition, virulence was evaluated on host from one geographic site, ignoring the effects of host evolution. Therefore, in the future, it is still necessary to interrogate the virulence evolutionary theory regarding multiple infections with mixed transmission modes using serial passage experiments (SPEs) in a controlled environment (45). Moreover, we categorized the foliar pathogens into vertical and horizontal transmission modes using only the sharing of seedborne fungi without verifying their within-host growth systematically one by one (see Fig. 2b). In fact, these foliar pathogens may transmit by a complex mode, e.g., the seedborne OTU1 fungi can also horizontally transmit (see Chen, Fang (46)), and some non-SFPs are expected to vertically transmit if fragments of stems and rhizomes are dispersed by the flooding during the expansion of *A. adenophora* (21). Indeed, many human, animal and plant diseases exhibit mixed vertical and horizontal transmission modes (5) and simultaneous infection by multiple species, as well as multiple genotypes of the same species (1, 2, 22, 23). Nonetheless, our investigation using a large number of pathogens from different species that coinfect a spreading invasive plant could be considered a virulence evolution experiment in a realistic environment. Surprisingly, the data verify that many predictions in classical models regarding multiple infections of strains from the same species (3) remain valid in such a complex system, in which the transmission mode (vertical vs horizontal) determines the virulence evolution trajectory and community dynamics of pathogens.” **(lines 295-314)**.

Reviewer #4:

I was asked to review the submission by Fang and colleagues with a specific focus on the statistical method used and the reporting of the data analysis. The analysis has clear merit and the main message is clearly of interest to a broad community of both fungi and plant biologist. Unfortunately there is a few points that need reworking to allow a thorough understanding of the study outcomes and potential replication of the analysis.

Response: Thank you very much. We have modified the description of data analysis and improved the description of the data results. Please find the details in the answers to the reviewers below.

**Part II – Major Issues: Key Experiments Required for Acceptance**

Reviewer #1: (No Response)

Reviewer #2: None.

Reviewer #3: Virulence is both a property of host evolution and pathogen evolution. The use of a single host to test virulence (before comparing to native plants) helps control for this, but it would be important to know where this host came from and how the virulence profiles among fungi differ across host lineages (as opposed to host species). Where the pathogen's profile sits on its current host seems to be just as important in confirming or testing hypotheses about virulence evolution. For instance, a pathogen can be avirulent towards its original host, but may remain virulent on a derived host if the host's susceptibility to adverse consequences of infection has also increased. I am not particularly convinced that the consideration of non-native plants suffices as a substitute for this.
Thus I think the authors should test virulence patterns on ideally hosts at multiple invasive populations and hosts at different extremes (20 yrs. v. 80 yrs env.) to ascertain the effect of hosts as well as pathogens (so that virulence evolution for instance actually is attenuated)

Response: Thank you very much for your concerns. We agreed “Virulence is both a property of host evolution and pathogen evolution”. Ideally, the virulence should be evaluated on the original host of the isolated strains; however, our research work involved 14 geographic sites, including 61 populations of *A. adenophora*, which across >1000 kilometers in geographic range; ~2705 strains were tested the virulence. In particular, because we focused on the multiple infections within leaf spot, we did not categorize the fungal strains based on plant individual in the sampling design thus missed the information of the corresponding original host. All of these factors make it impossible to test virulence on originally isolated host. We had to perform virulence on *A. adenophora* individuals grown in one geographic site (nearly 50 years of invasive history, see methods **(line 376)**), thus this really ignored the effects of host evolution. Fortunately, in another project, which attempts to explore if the host evolve to resist these pathogen attack, we selected two seedbrone pathogens to inoculate hosts from two history times (50y vs 80y), we did not observe the virulence difference on these two populations of host. We thus thought that host susceptibility to evolve was neglectable during this period of invasion. Nonetheless, in this revision we modified the presentation of our data based on an epidemiological investigation to alleviate this limitation; moreover, we also discussed this limitation in the conclusion paragraph **(lines 295-314)**.

Relatedly, I would like to see the community-structure account for source plant: it seems logical to me that pathogen communities from a leaf spot on the same plant are likely to show some structure. This can be done using a mixed-model with source plant as a random effect.

Response: Thank you very much for this concern. Firstly, we are sorry that missing some details in the methods causes a confusion on you. Actually, we did not characterize the pathogen community on a individual plant but at the geographical site level and at leaf spot level, here we pooled all leaf spots we collected from one geographic site (commonly involving multiple populations of *A. adenophora*) as a repeat for each history stage (commonly involving multiple geographic sites) of *A. adenophora* when characterizing the pathogen community of *A. adenophora* at geographic sits level; or we treated pathogen community from one leaf spot as a repeat for each history stage of *A. adenophora* when characterizing the pathogen community of *A. adenophora* at leaf spot level. We rewrote the “Data analysis” section, see details in **lines 404-419**.

Response to your concerns, in the revision we added a table for the statistics about the effects of invasive time and transmission mode on the pathogen community within leaf spot (Table 1), here you can see the transmission mode plays the most important role in structuring the pathogen community. Permutational analysis of variance (PERMANOVA) was used to test the differences in fungal community structure. Compare with mixed-model, this method can also show the degree to which factors contribute to microbial community variation (R^2^) and residual (see Table 1).

Reviewer #4: 1/ In the present submission, the order of introduction of the statistical methods is not consistent with presentation of the results. For example the use of a linera regression and the resulting test using an F statistic comes later than the description of the Mann-Whithney test. Make sure you are mapping rigorously the method description against the results for an easy understanding.

Response: Thank you very much. Based on your suggestions, we rewrote the “Data analysis” section, see details in **lines 404-419**.

2/ Althogether, most test used are quite cononical and well ackowledged and don't need excessive description in the materials and methods. However, when presenting a test statistic, make sure to use a specific and rigorous terminology. As an example when you describe in line 143 "pathogen communities were clustered by fungal transmission", you should write that "fungal transmission mode (seedborne pathogens (SPs) vs. non-seedborne pathogens (non-SPs) had a significant effect on pathogen community abundance? diversity/Shannon index?". Be precise about which exact dependant and independant variables you are using in the test, and make sure that the presentation of the results exactly matches the labels in the figures.

Response: Thank you very much. Based on your suggestions, we have modified some descriptions of the results, such as changed “diversity” to “Shannon diversity”, and changed “pathogen communities were clustered with invasive time” to “invasion time (80 years vs. 50 years vs. 20 years) had a significant effect on the pathogen community structure **(lines 151-152)**” and “pathogen communities were clustered by fungal transmission” to “the fungal transmission mode (SFPs vs. non-SFPs) impacted the pathogen community structure more than the invasion time **(lines 154-155)**”.

3/ Use a more appropriate term than % explanatory variables which I have not found elsewhere: you can say this variable explained X% of the variation for the shannon index (R2=Y, adj-R2=Z). Also report to figures as (Fig. S6b) for examples not (S6 Fig, panel b).

Response: Thank you very much. In this revised version, the results of redundancy analysis were deleted and replaced by a permutational analysis of variance (PERMANOVA) to verify the structural differences of microbial communities, see details in Table 1. In addition, we also have modified the representation of the “Supplementary materials” according to your suggestions.

**Part III – Minor Issues: Editorial and Data Presentation Modifications**

Reviewer #1:

The authors should do a better job of clearly defining terms, at their first use, for a general audience. These terms include multiple infections, horizontal and vertical transmission, and OTUs to name a few.

Response: Thank you very much. We defined these terms in the main text. For example, “multiple infections” was expressed as “… multiple infections, which are caused by multiple taxa of microbes” **(lines 54-55)**; “horizontal and vertical transmission” was expressed as “Most models assuming that parasites are transmitted horizontally (from the surrounding environment) predict that … **(line 61)**. If the parasites are transmitted vertically (from parent to offspring), … **(line 65)**”; “OTUs” was expressed as “A total of 2705 fungi (belonging to 451 operational taxonomic units, OTUs) … **(line 116)**”.

Overall, the figures are difficult to interpret. More descriptive figure legends would be helpful.

Response: Thanks. We have made appropriate changes for the figure legends. For example, we rewrote the legends for figure 1 and 2. We also modified other legends for clarification.

Reviewer #2:

I have two general critical comments:
1. The authors discuss evolutionary theory in the Introduction, and then return to it in the Conclusions, but a lot would be clarified by a figure that lists underlying hypotheses, the predictions, and results that either support reject. At the very least, set the paper by enumerating hypotheses and/or predictions in the Intro, and then explicitly refer back to them in the Conclusions.

Response: Thanks a lot for your suggestions. Following your suggestions, we set three major predictions by models in the last paragraph in the Introduction section as: “We expected that within a leaf spot of *A. adenophora*: (i) multiple fungal infections with mixed vertical and horizontal transmission are common; (ii) transmission mode can impact the pathogen community structure; and (iii) the virulence and community dynamics change in a different way between the vertically and horizontally transmitted pathogens along the invasive history of the host *A. adenophora* **(lines 104-108)**”. Then, we reminded readers if our data answered these three predictions in the conclusion paragraph as: “In conclusion, we employed the invasive plant *A. adenophora* as a model to verify many predictions of multiple infections of different fungal species with mixed vertical and horizontal transmission modes. This is the first investigation to reveal that multiple fungal infections with mixed vertical and horizontal transmission modes are common within the leaf spots of a natural plant system (the first expectation), and the vertical transmission mode determines the pathogen community structure (the second expectation). Our data also verified that the virulence and community dynamics changed in a different way between the vertically and horizontally transmitted pathogens and the invasive history of the host *A. adenophora* (the third expectation) **(lines 287-294)**.”

2. I think there are some issues with how this paper deals simplistically with the definition of “pathogen.” I think it is appropriate for this paper, but those shortcomings should be discussed. The authors performed detached leaf assays as a means to define virulence. This is fine, and it is done in many studies, but of course it is a limited tool. An isolate can cause lesions on a detached leaf and be of no consequence in an actual ecosystem. The authors mention also that many of the pathogens are known to grow asymptomatically in plants. It is well established that many, perhaps a majority, of plant pathogens can grow asymptomatically in plants, and thus fit a certain definition of ‘endophyte.’ We also know that such plant pathogenicity is often determined by “accessory” elements of the genome, that are potentially horizontally transmitted. It is possible that non-pathogenic and pathogenic forms of the same taxon may harbor a leaf spot, and that the non-pathogen could be converted to pathogen through some form of transfer. I do not believe that this study needs to deal directly with these issues, but it should acknowledge (add a paragraph to the Conclusions) that they have an effect on the results shown. I still believe the results support the conclusions.
Response: Thank you very much for your suggestions. We agreed your comments on the characteristics of plant pathogens. Indeed, an isolate can cause lesions on a detached leaf and be of no consequence in an actual ecosystem. To be avoid of this limitation of inoculating on detached leaves, actually, we tested the pathogenicity of all of these fungal strains by inoculation on mature and healthy leaves of plant individuals of *A. adenophora* grown in field rather than the detached leaves in the lab. We are sorry to confuse you because some details about the inoculation are missed in the methods.

In this version, we added some details in the methods to clarify: “The inoculation experiment was performed as previously reported to test the virulence of necrotrophic pathogens in tropical forests (53) with minor modifications. Briefly, fungi were grown on potato dextrose agar (PDA) for 7 d, and 6 mm^2^ agar discs with fungal mycelium were inoculated into mature and healthy leaves of *A. adenophora*. Small wounds were made by lightly touching the underside of the leaf with a sterilized toothpick, which resulted in a wound area of c. 0.2 cm^2^. The inoculum agar was pressed against the wound on the underside of the leaf using scotch tape, which was then clipped in place (Fig. S8). Each fungal strain was inoculated into five mature and healthy leaves from a single individual of *A. adenophora* to test pathogenicity. At 1 week after inoculation, leaves were harvested and photographed, and lesions (Fig. S8b) were measured **(lines 364-373).**” For the clarification, we also added a picture for performing disease experiment in supplementary (Fig. S8).

Considering some plant pathogens can grow asymptomatically in tissue for a while till produce symptom in a certain context, including abiotic and biotic environment (see: Garcia-Guzman G, Heil M. Life histories of hosts and pathogens predict patterns in tropical fungal plant diseases. New Phytologist. 2014;201(4):1106-20.). We added some sentences as: “Here, these seedborne foliar pathogens are typical necrotrophs, which can grow asymptomatically in plant tissue until becoming pathogenic under certain conditions (27, 28). Recently, a report indicated that for foliar fungi in forbs, vertical transmission from the mother plant to offspring via seeds was a widespread phenomenon (29). Furthermore, the evidence of microbial transmission routes from seeds to the phyllosphere was provided by growing oak seedlings in a microbe-free environment using an experimental culturing device (30). Thus, it is very interesting to determine how these seedborne fungi migrate to leaf tissue, where they cause disease, and how they interact with horizontally obtained fungi during the seedling development of *A. adenophora*.” **(Lines 201-209)**

I made a number of minor editorial suggestions on the Word document attached.

Response: Thank you very much for your editing. We modified manuscript as your suggestions. Here we also answered the several questions that you concerned one by one. We also invited a professional company (American Journal of Experts, AJE) for editing the language, and you can see the great improvements in the revised manuscripts.

Q: What are the pitfalls of this model?

Answer: Our research is not a real experimental evolution study, which commonly perform in a control manner in greenhouse. Although an observational study occurring in the nature may be a good alternative to a control experiment in current stage, the limitations of our system also exist. In this version, we made a substantial reversion to clarify this point: “However, our data were mainly based on epidemiological investigations and comparisons of different field populations of *A. adenophora*, without considering microhabitat differences or other potential selective factors across the sites. In addition, virulence was evaluated on host from one geographic site, ignoring the effects of host evolution. Therefore, in the future, it is still necessary to interrogate the virulence evolutionary theory regarding multiple infections with mixed transmission modes using serial passage experiments (SPEs) in a controlled environment (45). Moreover, we categorized the foliar pathogens into vertical and horizontal transmission modes using only the sharing of seedborne fungi without verifying their within-host growth systematically one by one (see Fig. 2b). In fact, these foliar pathogens may transmit by a complex mode, e.g., the seedborne OTU1 fungi can also horizontally transmit (see Chen, Fang (46)), and some non-SFPs are expected to vertically transmit if fragments of stems and rhizomes are dispersed by the flooding during the expansion of *A. adenophora* (21). Indeed, many human, animal and plant diseases exhibit mixed vertical and horizontal transmission modes (5) and simultaneous infection by multiple species, as well as multiple genotypes of the same species (1, 2, 22, 23). Nonetheless, our investigation using a large number of pathogens from different species that coinfect a spreading invasive plant could be considered a virulence evolution experiment in a realistic environment. Surprisingly, the data verify that many predictions in classical models regarding multiple infections of strains from the same species (3) remain valid in such a complex system, in which the transmission mode (vertical vs horizontal) determines the virulence evolution trajectory and community dynamics of pathogens.” **(Lines 295-314)**.

Q: Have these predictions been spelled out, as explicit hypotheses tested here?

Answer: Thank you very much. We set three major predictions by models in the last paragraph in the Introduction section as: “We expected that within a leaf spot of *A. adenophora*: (i) multiple fungal infections with mixed vertical and horizontal transmission are common; (ii) transmission mode can impact the pathogen community structure; and (iii) the virulence and community dynamics change in a different way between the vertically and horizontally transmitted pathogens along the invasive history of the host *A. Adenophora.*” **(Lines 104-108)**

Q: I do not think the pathogenicity experiments performed allow one to categorize the fungi as “pathogen” or “nopathogen.”

Answer: We added inoculation details in the methods, which expressed as “The inoculation experiment was performed as previously reported to test the virulence of necrotrophic pathogens in tropical forests (53) with minor modifications. Briefly, fungi were grown on potato dextrose agar (PDA) for 7 d, and 6 mm^2^ agar discs with fungal mycelium were inoculated into mature and healthy leaves of *A. adenophora*. Small wounds were made by lightly touching the underside of the leaf with a sterilized toothpick, which resulted in a wound area of c. 0.2 cm^2^. The inoculum agar was pressed against the wound on the underside of the leaf using scotch tape, which was then clipped in place (Fig. S8). Each fungal strain was inoculated into five mature and healthy leaves from a single individual of *A. adenophora* to test pathogenicity. At 1 week after inoculation, leaves were harvested and photographed, and lesions (Fig. S8b) were measured **(lines 364-373).**” We also provided a picture for performing disease experiment in supplementary (Fig. S8).

Considering some plant pathogens can grow asymptomatically in tissue for a while till produce symptom in a certain context, including abiotic and biotic environment (see: Garcia-Guzman G, Heil M. Life histories of hosts and pathogens predict patterns in tropical fungal plant diseases. New Phytologist. 2014;201(4):1106-20.). We added some sentences as: “Here, these seedborne foliar pathogens are typical necrotrophs, which can grow asymptomatically in plant tissue until becoming pathogenic under certain conditions (27, 28). Recently, a report indicated that for foliar fungi in forbs, vertical transmission from the mother plant to offspring via seeds was a widespread phenomenon (29). Furthermore, the evidence of microbial transmission routes from seeds to the phyllosphere was provided by growing oak seedlings in a microbe-free environment using an experimental culturing device (30). Thus, it is very interesting to determine how these seedborne fungi migrate to leaf tissue, where they cause disease, and how they interact with horizontally obtained fungi during the seedling development of *A. adenophora*.” **(Lines 201-209)**

Q: This is confusing – are you talking about amplicon libraries for NextGen sequencing?

Answer: We clarified this point in the methods, which expressed as “Then, to identify the fungi shared in foliar and seed samples of *A. adenophora*, the representative ITS sequences of each OTU from two libraries were pooled and aligned again.” **(Lines 385-387)**

Q: Pathogens vs. endophytes needs to be defined. A given fungus is not always pathogenic.

Answer: We discussed this point as “Here, these seedborne foliar pathogens are typical necrotrophs, which can grow asymptomatically in plant tissue until becoming pathogenic under certain conditions (27, 28). Recently, a report indicated that for foliar fungi in forbs, vertical transmission from the mother plant to offspring via seeds was a widespread phenomenon (29). Furthermore, evidence of microbial transmission routes from seeds to the phyllosphere was provided by growing oak seedlings in a microbe-free environment using an experimental culturing device (30). Thus, it is very interesting to determine how these seedborne fungi migrate to leaf tissue, where they cause disease, and how they interact with horizontally obtained fungi during the seedling development of *A. adenophora*.” **(Lines 201-209)**

Q: Their fitness depends on some degree of virulence as well.

Answer: We changed this sentence as “In contrast, vertically transmitted pathogens will decrease in virulence over time because their fitness primarily depends on hosts (3).” **(Lines 229-230)**

Reviewer #3:

Line 32-34: I did not understand what the authors mean here and found the wording confusing.

Response: Thank you very much. We changed it as “Our results demonstrate that the predictions of classical models for the virulence evolution of multiple infections are still valid in a complex realistic environment and highlight the impact of transmission mode on disease epidemics of foliar fungal pathogens. We also propose that seedborne fungi play an important role in structuring the foliar pathogen community from multiple infections within a leaf spot.” **(Lines 32-36)**

Line 39: check tense "occurring"

Response: Thanks. We changed “occurred” to “occurring”. **(Line 43)**

Lines 40-48 seem to just repeat the abstract.

Response: Thanks. We rewrote “Author Summary”. **(Lines 39-50)**

Line 80: "ecological time"

Response: Thanks. We changed “contemporary time” to “ecological time”. **(Line 80)**

Line 82: "same time scales"

Response: Thanks. We changed “at the same time” to “at the same time scales”. **(Line 82)**

Line 155-156: the higher virulence variation is an interesting result but I couldn't find much in the discussion about it.

Response: Thanks. We discussed it as “In addition, we also observed a linear relationship between the Shannon diversity of the pathogen and the variation in virulence within spots (Fig. 4a), as well as higher variation in the virulence within spots in young invasion fronts than in older invasion sites (Fig. 4b). These facts imply that, in the invasion front, sporadically distributed *A. adenophora* individuals are likely coinfected by diverse native pathogens with distinct but low virulence due to life-history trade-offs when infecting novel hosts (33); as more virulent strains evolve, they exclude weakly virulent strains and, in turn, cause decreased pathogen diversity within the leaf spot of *A. adenophora* over time.” **(Lines 222-229)**

Line 159-167, also methods 320-332: I never really understood what the role of the host range analysis in this paper was. It would seem a better test would be to look at different host strains/populations within the same lineage. See my summary/major comment.

Response: Thanks. For clarifying this point, in this version we changed this paragraph as: “A generalist strategy provides the parasite with more opportunities for transmission and persistence; however, specialists should be favored when hosts are abundant. Moreover, the evolution of virulence also depends on the host range; in theory, decreasing specificity can be associated with increasing virulence (34). We assume that in the invasion front, the native pathogen that can jump hosts to infect rare *A. adenophora* is most likely a generalist with a wide host range; therefore, this pathogen trends to narrow its host range with the increased host density in older geographic sites. In contrast, coinfection can offer opportunities for hybridization among different species to enlarge the host range (35); multiple infections within a leaf spot may broaden the host range for a host-specific seedborne fungus. However, both non-SFPs and SFPs showed no change in their host range and virulence to native plants (Fig. 5b, c). Because these pathogens changed in their virulence to the host *A. adenophora* (Fig. 4c), the results implied that the virulence of foliar fungal pathogens to their associated host might be a more plastic trait than host range, regardless of the transmission mode.” **(Lines 239-250)**

Lines 176, 195, 235: typo

Response: Thanks. We changed these. We also invited a professional company (American Journal of Experts, AJE) for editing our manuscript.

Line 185-187: I think it would help the authors to remind the reader where this is in the results.

Response: Thanks. We changed this sentence and marked the figure number, which was expressed as “More interestingly, we found that 25.3% of foliar pathogens of *A. adenophora* were related to seedborne fungi (Fig. 2a, File S1), which primarily determine the assembly of pathogen communities within a leaf spot (Fig. 3b).” **(Lines 193-195)**

Line 202-203: I'm not really sure this conclusion is warranted.

Response: Thanks. We deleted this conclusion.

Line 220-223: this feels too speculative.

Response: Thanks. We deleted the speculative sentence, we rewrote this sentence as “Because less virulent pathogens are competitively inferior in the case of mixed infections (3), it is interesting to explore whether the virulence of SFPs will constantly decrease to a level lower than that of non-SFPs or to a balance point in a certain amount of time with the delayed invasive history.” **(Lines 235-238)**

Lines 238-240: I think this illustrates the role of demography nicely, but there is little discussion of that in the paper.

Response: Thanks. We modified this paragraph as: “A generalist strategy provides the parasite with more opportunities for transmission and persistence; however, specialists should be favored when hosts are abundant. Moreover, the evolution of virulence also depends on the host range; in theory, decreasing specificity can be associated with increasing virulence (34). We assume that in the invasion front, the native pathogen that can jump hosts to infect rare *A. adenophora* is most likely a generalist with a wide host range; therefore, this pathogen trends to narrow its host range with the increased host density in older geographic sites. In contrast, coinfection can offer opportunities for hybridization among different species to enlarge the host range (35); multiple infections within a leaf spot may broaden the host range for a host-specific seedborne fungus. However, both non-SFPs and SFPs showed no change in their host range and virulence to native plants (Fig. 5b, c). Because these pathogens changed in their virulence to the host *A. adenophora* (Fig. 4c), the results implied that the virulence of foliar fungal pathogens to their associated host might be a more plastic trait than host range, regardless of the transmission mode.” **(Lines 239-250)**

Lines 343-345: I confess to not being an expert in fungal pathogens of plants. Do seed-borne pathogens necessarily go on to infect their host and form leaf spots? How predictive is being found in the seed predictive of a pathogen being found in the same individual at a later developmental stage? How do we know this pathogen wasn't horizontally transmitted from another individual who happened to get it vertically? I think a short sentence or two giving the basics of these sorts of issues will reassure readers working in other systems of the appropriateness of the SP/non-SP classification.0

Response: Thanks for your concerns. These questions you mentioned here indeed are what the plant pathologists and microbial ecologists have concerned for a long time. However, the experimental works answering these questions are just beginning. Traditionally, plants have been thought to receive foliar pathogens from surrounding environments (horizontally transmitted) without vertically transmitted pathogens because the seeds are microbe-free. However, increasing evidences have found that seeds commonly contain diverse microbes. Currently, these works mainly are about crops, in which seedborne fungi can transfer to seedlings to cause disease (see Basak AB and Min WL. Prevalence and Transmission of Seed-Borne Fungi of Maize Grown in a Farm of Korea. Mycobiology. 2002;30(1):47-50.). Till in recent, scientists have concerned such a phenomenon in the wild species in the ecosystem, e.g., a report indicated that, for foliar fungi in forbs, the vertical transmission from mother plant to offspring, via seeds, was a widespread phenomenon (Hodgson S, Cates CD, Hodgson J, et al. Vertical transmission of fungal endophytes is widespread in forbs. Ecology and Evolution 2014;4(8):1199–1208). Excitingly, just at the moment we revised our manuscript, a report provided the evidence of microbial transmission routes from seed to phyllosphere by growing oak seedlings in a microbe-free environment using an ingenious experimental culturing device (see Abdelfattah A, Wisniewski M, Schena L, Tack AJM. Experimental evidence of microbial inheritance in plants and transmission routes from seed to phyllosphere and root. Environ Microbiol. 2021.). Therefore, we believe that these pioneer works will inspire more research works for exploring the evidences that some foliar pathogens are from seedborne fungi and thus can change the traditional knowledge about plant foliar disease.

In this version, we added information for this interesting topic as: “Here, these seedborne foliar pathogens are typical necrotrophs, which can grow asymptomatically in plant tissue until becoming pathogenic under certain conditions (27, 28). Recently, a report indicated that for foliar fungi in forbs, vertical transmission from the mother plant to offspring via seeds was a widespread phenomenon (29). Furthermore, evidence of microbial transmission routes from seeds to the phyllosphere was provided by growing oak seedlings in a microbe-free environment using an experimental culturing device (30). Thus, it is very interesting to determine how these seedborne fungi migrate to leaf tissue, where they cause disease, and how they interact with horizontally obtained fungi during the seedling development of *A. adenophora*.” **(Lines 201-209)**

Reviewer #4:

You do not necessarily need to describe the libraries you used to draw the graphs and plots, these are not the critical part of the analysis. However, be mindful of introducing the sample sizes when making comparison, either through the reporting of degrees of freedoms when reporting a statistical test, or putting n=100 on the figure labels.

Response: Thanks. We rewrote the “Data analysis” section, see details in **lines 404-419**. Moreover, we added the information about sample sizes in the figures or figure legends (Fig. 3, 4, 5).

Finally, I do think the manuscript would benefit from a professional English editing service to make sure there is no miscommunication. For example, when you use the verb "overlook" in line 312, you mean the exact contrary.

Response: Thanks. We changed this as “without considering” **(line 354)**. As your suggestions, we invited a professional company (American Journal of Experts, AJE) for editing our manuscript to improve language quality.
